# First records of 31 species of butterflies and moths (Lepidoptera) in Cameroon, with remarks on their elevational ranges

**DOI:** 10.3897/BDJ.8.e50543

**Published:** 2020-03-05

**Authors:** Sylvain Delabye, Vincent Maicher, Szabolcs Sáfián, Pavel Potocký, Jan E.J. Mertens, Łukasz Przybyłowicz, Mercy Murkwe, Ishmeal N. Kobe, Eric B. Fokam, Štěpán Janeček, Robert Tropek

**Affiliations:** 1 Biology Centre, Czech Academy of Science, Institute of Entomology, Branisovska 31, CZ-37005 Ceske Budejovice, Czech Republic Biology Centre, Czech Academy of Science, Institute of Entomology Branisovska 31, CZ-37005 Ceske Budejovice Czech Republic; 2 Faculty of Science, University of South Bohemia, Branisovska 1760, CZ-37005 Ceske Budejovice, Czech Republic Faculty of Science, University of South Bohemia Branisovska 1760, CZ-37005 Ceske Budejovice Czech Republic; 3 Department of Ecology, Faculty of Science, Charles University, Vinicna 7, CZ-12844 Prague, Czech Republic Department of Ecology, Faculty of Science, Charles University Vinicna 7, CZ-12844 Prague Czech Republic; 4 Institute of Silviculture and Forest Protection, Faculty of Forestry, University of West Hungary, Bajcsy-Zsilinszky utca 4, H-9400 Sopron, Hungary Institute of Silviculture and Forest Protection, Faculty of Forestry, University of West Hungary Bajcsy-Zsilinszky utca 4, H-9400 Sopron Hungary; 5 Biodiversity Inventory for Conservation NPO (BINCO), Walmersumstraat 44, 3380 Glabbeek, Belgium Biodiversity Inventory for Conservation NPO (BINCO) Walmersumstraat 44, 3380 Glabbeek Belgium; 6 Institute of Systematics and Evolution of Animals, Polish Academy of Sciences, Sławkowska 17, PL-31-016 Krakow, Poland Institute of Systematics and Evolution of Animals, Polish Academy of Sciences Sławkowska 17, PL-31-016 Krakow Poland; 7 Department of Zoology and Animal Physiology, Faculty of Science, University of Buea, P.O. Box 63, Buea, Cameroon Department of Zoology and Animal Physiology, Faculty of Science, University of Buea P.O. Box 63, Buea Cameroon

**Keywords:** Afrotropics, bait-trapping, Bamenda Highlands, faunistics, light-trapping, Mount Cameroon

## Abstract

**Background:**

The biodiversity of West and Central Africa is understudied, including butterflies and moths (Lepidoptera). Cameroon, through its position in between few biogeographic regions and diversity of habitats, is an important hotspot of lepidopteran diversity. However, the country also ranks low when it comes to local biodiversity knowledge. During our long-term ecological projects in the Cameroonian part of the Gulf of Guinea Highlands, we collected rich material of butterflies and moths, including a number of interesting faunistic records.

**New information:**

In this study, we report 31 species of butterflies and moths which have not yet been recorded in Cameroon. These species comprised eight new genera records for the country. In many cases, our records represented an important extension of the species’ known distribution, including ten species whose distribution ranges extended into the Guinean biogeographic region. We also comment on the species’ elevational distribution ranges on Mount Cameroon where most of our records originated. Additionally, we confirm the presence of a butterfly *Telchinia encedena*, after more than a century since its first and so far its only record in Cameroon.

## Introduction

West and Central Sub-Saharan Africa belong to the areas with the lowest knowledge on regional biodiversity on the continent. Although almost 3,000 taxa of moths (De Prins and De Prins 2019) and almost 1,600 taxa of butterflies (Williams 2018) have been reported from Cameroon so far, much higher diversity of these groups can be expected, considering the country’s high habitat heterogeneity. The Gulf of Guinea Highlands is an important montane range on the borders of Nigeria and Cameroon (i.e. at the edge of the Guinean and Congolian biogeographic regions). It represents the only large montane area in the region and is recognised as a biodiversity hotspot with high endemism for numerous taxa (Myers et al. 2000), including butterflies and moths (Larsen 2005, Ustjuzhanin et al. 2018). However, the biodiversity of this montane range is still relatively unexplored and comprehensive studies of Lepidoptera are still under-represented.

Several recent ecological studies on lepidopteran communities in the Gulf of Guinea Highlands (e.g. Tropek and Konvička 2010, Maicher et al. 2018, Maicher et al. 2020) collected an extensive number of butterflies and moths, including several newly described species (e.g. Yakovlev and Sáfián 2016, Ustjuzhanin et al. 2018, Sáfián and Tropek 2016, Sáfián et al. 2019), as well as new country records already published during earlier stages of the projects (e.g. Tropek et al. 2013, Tropek et al. 2015, Maicher et al. 2016, Ustjuzhanin et al. 2018, Przybyłowicz et al. 2019b). The large amount of collected material still holds new species of general faunistic or taxonomic interest. In this study, we report 31 species of butterflies and moths recorded in Cameroon for the first time. In some cases, these records significantly extended the species’ known geographical ranges. These are supplemented by a rediscovery of a butterfly species in Cameroon after more than a century.

## Materials and methods

**Figure 1.**
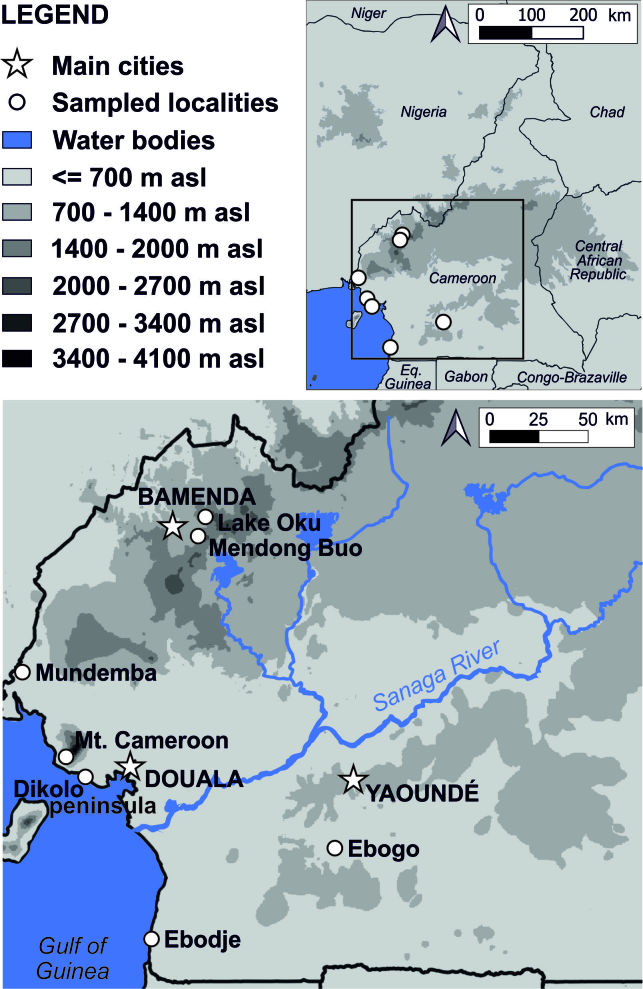
Map of Cameroon with the study sites and the Sanaga river considered as a border between the Guinean and Congolian biogeographic regions.

All reported butterfly and moth specimens were collected between 2008 and 2017 in Cameroon (Fig. 1).

Nine sampled localities lie in the Mount Cameroon region, Fako Division, Southwest Region, Cameroon. Seven of these localities are on the south-western slope of Mount Cameroon inside the Mount Cameroon National Park, in tropical rainforests at different elevations. These represent mosaics of primary and secondary lowland forests (Bamboo Camp, 350 m a.s.l.; Drink Gari camp, 650 m a.s.l.), through upland (PlanteCam camp, 1,100 m a.s.l.) and submontane (Crater Lake camp, 1,450 m a.s.l.) forests locally disturbed by elephants, to montane forests (Elephant Camp, 1,850 m a.s.l.; Mapanja camp, 1,850 m a.s.l.; Mann’s Spring, 2,200 m a.s.l.) (Fig. 2b, c, d, e). The two remaining localities in the Mount Cameroon region are situated in a coastal forest (Fig. 2a) of the Bimbia-Bonadikombo Community Forest (Dikolo Peninsula camp, 30 m a.s.l.; Ferenc et al. 2018) and in a heavily disturbed lowland forest on a lower hill with a cell tower close to the Chop Farm junction, Bimbia village (Radio Hill, 220 m a.s.l.).

The other two localities are situated in the Bamenda Highlands, Northwest Region, Cameroon. Mendong Buo, ca. 5 km south-east of Big Babanki, represents a mosaic of montane forest remnants, forest clearings dominated by *Pteridium aquilinum*, submontane grasslands maintained by cattle grazing and species‐rich scrub vegetation along streams (Tropek and Konvička 2010; Fig. 2f). Lake Oku represents a primary montane forest along the crater lake shore, on the southern slopes of Mount Oku.

The last three localities are in disturbed lowland forests across the country. Mundemba represents heavily disturbed secondary regrowth south of Mundemba, close to the Korup NP, Southwest Region. Ebogo is a disturbed lowland rainforest in the Ebogo Ecotouristic Site, ca. 80 km south of Yaounde, Centre Region. Lastly, close to Ebodje, South Region, butterflies were recorded in a secondary lowland forest.

All moths were attracted to light (see Maicher et al. 2020 for the detailed protocol). Butterflies were collected by traps baited with fermented mashed bananas (*Euphaedra temeraria*, see Maicher et al. 2020 for details) or by a butterfly net (all other butterfly species).

Nomenclature and distribution of the reported species were based on the AfroMoths online database (De Prins and De Prins 2019) for moths and on Williams (2018) for butterflies. The biogeographic regions nomenclature follows Linder et al. (2012). The voucher material is deposited in the Nature Education Centre of the Jagiellonian University in Kraków, Poland and in the Biology Centre, Czech Academy of Sciences, České Budějovice, Czechia.

## Taxon treatments

### Anapisa holobrunnea

(Talbot, 1932)

CD76542F-C73E-5AB0-BB38-C5FA53AE9132

Erebidae , Arctiinae

#### Materials

- **Type status:** Other material. **Occurrence:** individualCount: 1; sex: male; lifeStage: adult; **Taxon:** scientificName: Anapisa holobrunnea (Talbot, 1932); **Location:** continent: Africa; country: Cameroon; stateProvince: Southwest Region; locality: Dikolo Peninsula, Bimbia-Bonadikombo Community Forest; verbatimElevation: 30 m; decimalLatitude: 03.9818; decimalLongitude: 09.2625; **Identification:** identifiedBy: Łukasz Przybyłowicz; dateIdentified: 2017; **Event:** samplingProtocol: Light catching; eventDate: 13/10/2017; habitat: Coastal forest; **Record Level:** type: PhysicalObject; institutionID: http://grbio.org/cool/8t1f-g2z6; institutionCode: ZMJU; basisOfRecord: PreservedSpecimen

#### Distribution

**Figure 3.**
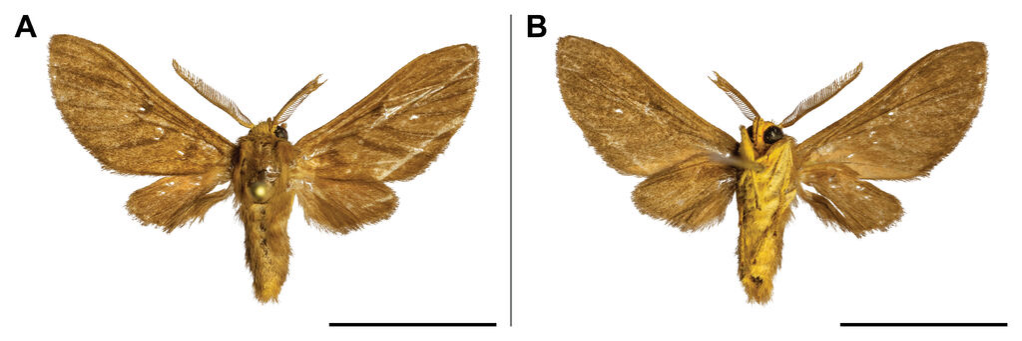
*Anapisa holobrunnea* Tams, 1932. **A.** dorsal view; **B.** ventral view. The scale bar represents 1 cm.

*A. holobrunnea* was previously reported from Ghana and Guinea only (Przybyłowicz and Bąkowski 2011) and, therefore, it was considered as an endemic to the western part of the Guinean subregion. Our record has enlarged its known range to over 1,000 km to the east. It has also evidenced the species’ presence in the eastern part of the Guinean region. In the Mount Cameroon region, the single individual was collected in the lowest locality (30 m a.s.l.) (Fig. 3).

### Anapisa metarctioides

(Hampson, 1907)

B8BD6C21-D4F3-539F-B454-814BDFE3A2FB

Erebidae , Arctiinae

#### Materials

- **Type status:** Other material. **Occurrence:** individualCount: 5; sex: males; lifeStage: adult; **Taxon:** scientificName: Anapisa metarctioides (Hampson, 1907); **Location:** continent: Africa; country: Cameroon; stateProvince: Southwest Region; locality: PlanteCam Camp, Mount Cameroon; verbatimElevation: 1,100 m; decimalLatitude: 04.1175; decimalLongitude: 09.0709; **Identification:** identifiedBy: Łukasz Przybyłowicz; dateIdentified: 2017; **Event:** samplingProtocol: Light catching; eventDate: 13/12/2014; habitat: Upland forest locally disturbed by elephants; **Record Level:** type: PhysicalObject; institutionID: http://grbio.org/cool/8t1f-g2z6; institutionCode: ZMJU; basisOfRecord: PreservedSpecimen
- **Type status:** Other material. **Occurrence:** individualCount: 1; sex: male; lifeStage: adult; **Taxon:** scientificName: Anapisa metarctioides (Hampson, 1907); **Location:** continent: Africa; country: Cameroon; stateProvince: Southwest Region; locality: PlanteCam Camp, Mount Cameroon; verbatimElevation: 1,100 m; decimalLatitude: 04.1175; decimalLongitude: 09.0709; **Identification:** identifiedBy: Łukasz Przybyłowicz; dateIdentified: 2017; **Event:** samplingProtocol: Light catching; eventDate: 13/04/2015; habitat: Upland forest locally disturbed by elephants; **Record Level:** type: PhysicalObject; institutionID: http://grbio.org/cool/8t1f-g2z6; institutionCode: ZMJU; basisOfRecord: PreservedSpecimen
- **Type status:** Other material. **Occurrence:** individualCount: 5; sex: males; lifeStage: adult; **Taxon:** scientificName: Anapisa metarctioides (Hampson, 1907); **Location:** continent: Africa; country: Cameroon; stateProvince: Southwest Region; locality: PlanteCam Camp, Mount Cameroon; verbatimElevation: 1,100 m; decimalLatitude: 04.1175; decimalLongitude: 09.0709; **Identification:** identifiedBy: Łukasz Przybyłowicz; dateIdentified: 2017; **Event:** samplingProtocol: Light catching; eventDate: 01/02/2016; habitat: Upland forest locally disturbed by elephants; **Record Level:** type: PhysicalObject; institutionID: http://grbio.org/cool/8t1f-g2z6; institutionCode: ZMJU; basisOfRecord: PreservedSpecimen
- **Type status:** Other material. **Occurrence:** individualCount: 10; sex: males; lifeStage: adult; **Taxon:** scientificName: Anapisa metarctioides (Hampson, 1907); **Location:** continent: Africa; country: Cameroon; stateProvince: Southwest Region; locality: Crater Lake, Mount Cameroon; verbatimElevation: 1,450 m; decimalLatitude: 04.1443; decimalLongitude: 09.0717; **Identification:** identifiedBy: Łukasz Przybyłowicz; dateIdentified: 2017; **Event:** samplingProtocol: Light catching; eventDate: 24/11/2016; habitat: Submontane forest locally disturbed by elephants; **Record Level:** type: PhysicalObject; institutionID: http://grbio.org/cool/8t1f-g2z6; institutionCode: ZMJU; basisOfRecord: PreservedSpecimen
- **Type status:** Other material. **Occurrence:** individualCount: 8; sex: males; lifeStage: adult; **Taxon:** scientificName: Anapisa metarctioides (Hampson, 1907); **Location:** continent: Africa; country: Cameroon; stateProvince: Southwest Region; locality: Crater Lake, Mount Cameroon; verbatimElevation: 1,450 m; decimalLatitude: 04.1443; decimalLongitude: 09.0717; **Identification:** identifiedBy: Łukasz Przybyłowicz; dateIdentified: 2017; **Event:** samplingProtocol: Light catching; eventDate: 20/02/2017; habitat: Submontane forest locally disturbed by elephants; **Record Level:** type: PhysicalObject; institutionID: http://grbio.org/cool/8t1f-g2z6; institutionCode: ZMJU; basisOfRecord: PreservedSpecimen
- **Type status:** Other material. **Occurrence:** individualCount: 4; sex: males; lifeStage: adult; **Taxon:** scientificName: Anapisa metarctioides (Hampson, 1907); **Location:** continent: Africa; country: Cameroon; stateProvince: Southwest Region; locality: Crater Lake, Mount Cameroon; verbatimElevation: 1,450 m; decimalLatitude: 04.1443; decimalLongitude: 09.0717; **Identification:** identifiedBy: Łukasz Przybyłowicz; dateIdentified: 2017; **Event:** samplingProtocol: Light catching; eventDate: 28/04/2017; habitat: Submontane forest locally disturbed by elephants; **Record Level:** type: PhysicalObject; institutionID: http://grbio.org/cool/8t1f-g2z6; institutionCode: ZMJU; basisOfRecord: PreservedSpecimen
- **Type status:** Other material. **Occurrence:** individualCount: 7; sex: males; lifeStage: adult; **Taxon:** scientificName: Anapisa metarctioides (Hampson, 1907); **Location:** continent: Africa; country: Cameroon; stateProvince: Southwest Region; locality: Elephant Camp, Mount Cameroon; verbatimElevation: 1,850 m; decimalLatitude: 04.1453; decimalLongitude: 09.0870; **Identification:** identifiedBy: Łukasz Przybyłowicz; dateIdentified: 2017; **Event:** samplingProtocol: Light catching; eventDate: 21/11/2014; habitat: Montane forest locally disturbed by elephants; **Record Level:** type: PhysicalObject; institutionID: http://grbio.org/cool/8t1f-g2z6; institutionCode: ZMJU; basisOfRecord: PreservedSpecimen
- **Type status:** Other material. **Occurrence:** individualCount: 12; sex: males; lifeStage: adult; **Taxon:** scientificName: Anapisa metarctioides (Hampson, 1907); **Location:** continent: Africa; country: Cameroon; stateProvince: Southwest Region; locality: Elephant Camp, Mount Cameroon; verbatimElevation: 1,850 m; decimalLatitude: 04.1453; decimalLongitude: 09.0870; **Identification:** identifiedBy: Łukasz Przybyłowicz; dateIdentified: 2017; **Event:** samplingProtocol: Light catching; eventDate: 20/02/2017; habitat: Montane forest locally disturbed by elephants; **Record Level:** type: PhysicalObject; institutionID: http://grbio.org/cool/8t1f-g2z6; institutionCode: ZMJU; basisOfRecord: PreservedSpecimen
- **Type status:** Other material. **Occurrence:** individualCount: 9; sex: 8 males, 1 female; lifeStage: adult; **Taxon:** scientificName: Anapisa metarctioides (Hampson, 1907); **Location:** continent: Africa; country: Cameroon; stateProvince: Southwest Region; locality: Elephant Camp, Mount Cameroon; verbatimElevation: 1,850 m; decimalLatitude: 04.1453; decimalLongitude: 09.0870; **Identification:** identifiedBy: Łukasz Przybyłowicz; dateIdentified: 2017; **Event:** samplingProtocol: Light catching; eventDate: 22/04/2017; habitat: Montane forest locally disturbed by elephants; **Record Level:** type: PhysicalObject; institutionID: http://grbio.org/cool/8t1f-g2z6; institutionCode: ZMJU; basisOfRecord: PreservedSpecimen
- **Type status:** Other material. **Occurrence:** individualCount: 1; sex: male; lifeStage: adult; **Taxon:** scientificName: Anapisa metarctioides (Hampson, 1907); **Location:** continent: Africa; country: Cameroon; stateProvince: Southwest Region; locality: Mann’s Spring, Mount Cameroon; verbatimElevation: 2,200 m; decimalLatitude: 04.1428; decimalLongitude: 09.1225; **Identification:** identifiedBy: Łukasz Przybyłowicz; dateIdentified: 2017; **Event:** samplingProtocol: Light catching; eventDate: 09/11/2016; habitat: Montane forest close to the timberline; **Record Level:** type: PhysicalObject; institutionID: http://grbio.org/cool/8t1f-g2z6; institutionCode: ZMJU; basisOfRecord: PreservedSpecimen

#### Distribution

**Figure 4.**
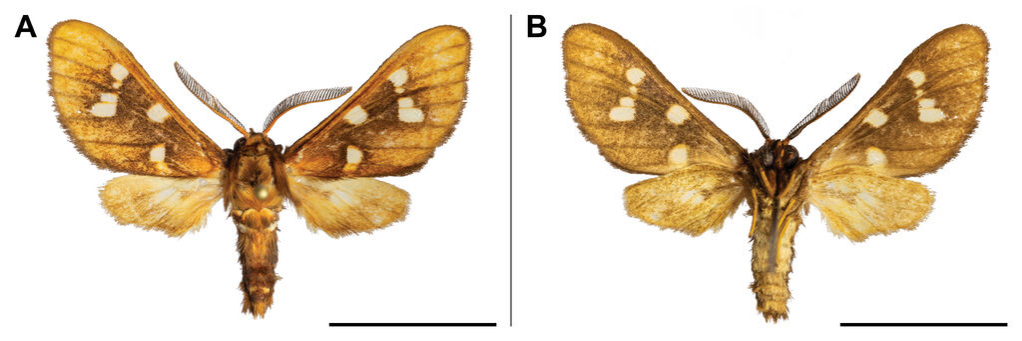
*Anapisa metarctioides* (Hampson, 1907). **A.** dorsal view; **B.** ventral view. The scale bar represents 1 cm.

This species was known from Kenya, Uganda, Rwanda and the Democratic Republic of Congo. Our record on Mount Cameroon has extended its western distribution and has evidenced the species from the Guinean biogeographic region. On Mount Cameroon, it is restricted to above 1,100 m a.s.l. (Fig. 4).

### Archithosia makomensis

(Strand, 1912)

600C8FCC-FD19-5F1D-95DA-C10B04A44480

Erebidae , Arctiinae

#### Materials

- **Type status:** Other material. **Occurrence:** individualCount: 1; sex: male; lifeStage: adult; **Taxon:** scientificName: Archithosia makomensis (Strand, 1912); **Location:** continent: Africa; country: Cameroon; stateProvince: Southwest Region; locality: Elephant Camp, Mount Cameroon; verbatimElevation: 1,850 m; decimalLatitude: 04.1453; decimalLongitude: 09.0870; **Identification:** identifiedBy: Łukasz Przybyłowicz; dateIdentified: 2017; **Event:** samplingProtocol: Light catching; eventDate: 26/04/2017; habitat: Montane forest locally disturbed by elephants; **Record Level:** type: PhysicalObject; institutionID: http://grbio.org/cool/8t1f-g2z6; institutionCode: ZMJU; basisOfRecord: PreservedSpecimen
- **Type status:** Other material. **Occurrence:** individualCount: 1; sex: male; lifeStage: adult; **Taxon:** scientificName: Balacra compsa (Jordan, 1904); **Location:** continent: Africa; country: Cameroon; stateProvince: Southwest Region; locality: Crater Lake, Mount Cameroon; verbatimElevation: 1,450 m; decimalLatitude: 04.1443; decimalLongitude: 09.0717; **Identification:** identifiedBy: Łukasz Przybyłowicz; dateIdentified: 2017; **Event:** samplingProtocol: Light catching; eventDate: 26/04/2017; habitat: Submontane forest locally disturbed by elephants; **Record Level:** type: PhysicalObject; institutionID: http://grbio.org/cool/8t1f-g2z6; institutionCode: ZMJU; basisOfRecord: PreservedSpecimen
- **Type status:** Other material. **Occurrence:** individualCount: 1; sex: male; lifeStage: adult; **Taxon:** scientificName: Balacra compsa (Jordan, 1904); **Location:** continent: Africa; country: Cameroon; stateProvince: Southwest Region; locality: Elephant Camp, Mount Cameroon; verbatimElevation: 1,850 m; decimalLatitude: 04.1453; decimalLongitude: 09.0870; **Identification:** identifiedBy: Łukasz Przybyłowicz; dateIdentified: 2017; **Event:** samplingProtocol: Light catching; eventDate: 20/11/2014; habitat: Montane forest locally disturbed by elephants; **Record Level:** type: PhysicalObject; institutionID: http://grbio.org/cool/8t1f-g2z6; institutionCode: ZMJU; basisOfRecord: PreservedSpecimen
- **Type status:** Other material. **Occurrence:** individualCount: 1; sex: male; lifeStage: adult; **Taxon:** scientificName: Balacra compsa (Jordan, 1904); **Location:** continent: Africa; country: Cameroon; stateProvince: Southwest Region; locality: Elephant Camp, Mount Cameroon; verbatimElevation: 1,850 m; decimalLatitude: 04.1453; decimalLongitude: 09.0870; **Identification:** identifiedBy: Łukasz Przybyłowicz; dateIdentified: 2017; **Event:** samplingProtocol: Light catching; eventDate: 20/04/2017; habitat: Montane forest locally disturbed by elephants; **Record Level:** type: PhysicalObject; institutionID: http://grbio.org/cool/8t1f-g2z6; institutionCode: ZMJU; basisOfRecord: PreservedSpecimen

#### Distribution

**Figure 5.**
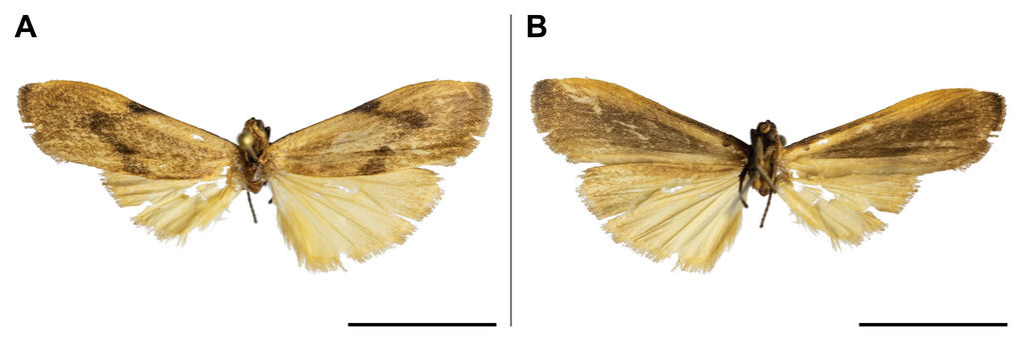
*Archithosia makomensis* (Strand, 1912). **A.** dorsal view; **B.** ventral view. The scale bar represents 1 cm.

This species was known from Ghana, Nigeria and Equatorial Guinea. Hence, its distribution in Cameroon was thus expected, although never reported before. The only specimen was caught in montane forest (1,850 m a.s.l.) (Fig. 5).

### Balacra compsa

(Jordan, 1904)

B51C70E6-D201-51FB-9857-20369831F8C2

Erebidae , Arctiinae

#### Materials

- **Type status:** Other material. **Occurrence:** individualCount: 1; sex: male; lifeStage: adult; **Taxon:** scientificName: Balacra compsa (Jordan, 1904); **Location:** continent: Africa; country: Cameroon; stateProvince: Southwest Region; locality: Crater Lake, Mount Cameroon; verbatimElevation: 1,450 m; decimalLatitude: 04.1443; decimalLongitude: 09.0717; **Identification:** identifiedBy: Łukasz Przybyłowicz; dateIdentified: 2017; **Event:** samplingProtocol: Light catching; eventDate: 26/04/2017; habitat: Submontane forest locally disturbed by elephants; **Record Level:** type: PhysicalObject; institutionID: http://grbio.org/cool/8t1f-g2z6; institutionCode: ZMJU; basisOfRecord: PreservedSpecimen
- **Type status:** Other material. **Occurrence:** individualCount: 1; sex: male; lifeStage: adult; **Taxon:** scientificName: Balacra compsa (Jordan, 1904); **Location:** continent: Africa; country: Cameroon; stateProvince: Southwest Region; locality: Elephant Camp, Mount Cameroon; verbatimElevation: 1,850 m; decimalLatitude: 04.1453; decimalLongitude: 09.0870; **Identification:** identifiedBy: Łukasz Przybyłowicz; dateIdentified: 2017; **Event:** samplingProtocol: Light catching; eventDate: 20/11/2014; habitat: Montane forest locally disturbed by elephants; **Record Level:** type: PhysicalObject; institutionID: http://grbio.org/cool/8t1f-g2z6; institutionCode: ZMJU; basisOfRecord: PreservedSpecimen
- **Type status:** Other material. **Occurrence:** individualCount: 1; sex: male; lifeStage: adult; **Taxon:** scientificName: Balacra compsa (Jordan, 1904); **Location:** continent: Africa; country: Cameroon; stateProvince: Southwest Region; locality: Elephant Camp, Mount Cameroon; verbatimElevation: 1,850 m; decimalLatitude: 04.1453; decimalLongitude: 09.0870; **Identification:** identifiedBy: Łukasz Przybyłowicz; dateIdentified: 2017; **Event:** samplingProtocol: Light catching; eventDate: 20/04/2017; habitat: Montane forest locally disturbed by elephants; **Record Level:** type: PhysicalObject; institutionID: http://grbio.org/cool/8t1f-g2z6; institutionCode: ZMJU; basisOfRecord: PreservedSpecimen

#### Distribution

**Figure 6.**
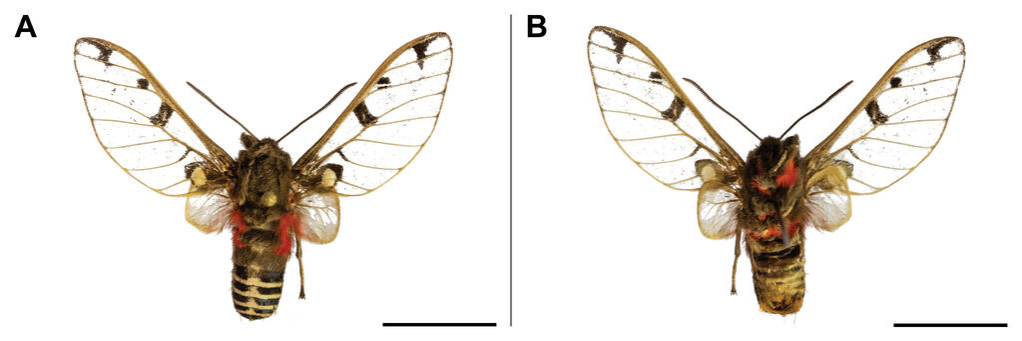
*Balacra compsa* (Jordan, 1904). **A.** dorsal view; **B.** ventral view. The scale bar represents 1 cm.

This species was previously known from the Congolian and Shaba biogeographic regions (Przybyłowicz 2013). Our record extended its distribution to the Guinean biogeographic region. We collected it only in submontane and montane forests (1,450 and 1,850 m a.s.l.) (Fig. 6).

### Daphaenisca inexpectata

(Durante & Zangrilli, 2016)

893A9535-43F1-512E-8D72-442F1BA7CA5D

Erebidae , Arctiinae

#### Materials

- **Type status:** Other material. **Occurrence:** individualCount: 14; sex: males; lifeStage: adult; **Taxon:** scientificName: Daphaenisca inexpectata (Durante & Zangrilli, 2016); **Location:** continent: Africa; country: Cameroon; stateProvince: Southwest Region; locality: Bamboo Camp, Mount Cameroon; verbatimElevation: 350 m; decimalLatitude: 04.0899; decimalLongitude: 09.0517; **Identification:** identifiedBy: Łukasz Przybyłowicz; dateIdentified: 2017; **Event:** samplingProtocol: Light catching; eventDate: 16/12/2014; habitat: Lowland forest disturbed by historical selective logging; **Record Level:** type: PhysicalObject; institutionID: http://grbio.org/cool/8t1f-g2z6; institutionCode: ZMJU; basisOfRecord: PreservedSpecimen
- **Type status:** Other material. **Occurrence:** individualCount: 26; sex: males; lifeStage: adult; **Taxon:** scientificName: Daphaenisca inexpectata (Durante & Zangrilli, 2016); **Location:** continent: Africa; country: Cameroon; stateProvince: Southwest Region; locality: Bamboo Camp, Mount Cameroon; verbatimElevation: 350 m; decimalLatitude: 04.0899; decimalLongitude: 09.0517; **Identification:** identifiedBy: Łukasz Przybyłowicz; dateIdentified: 2017; **Event:** samplingProtocol: Light catching; eventDate: 20/04/2015; habitat: Lowland forest disturbed by historical selective logging; **Record Level:** type: PhysicalObject; institutionID: http://grbio.org/cool/8t1f-g2z6; institutionCode: ZMJU; basisOfRecord: PreservedSpecimen
- **Type status:** Other material. **Occurrence:** individualCount: 9; sex: males; lifeStage: adult; **Taxon:** scientificName: Daphaenisca inexpectata (Durante & Zangrilli, 2016); **Location:** continent: Africa; country: Cameroon; stateProvince: Southwest Region; locality: Bamboo Camp, Mount Cameroon; verbatimElevation: 350 m; decimalLatitude: 04.0899; decimalLongitude: 09.0517; **Identification:** identifiedBy: Łukasz Przybyłowicz; dateIdentified: 2017; **Event:** samplingProtocol: Light catching; eventDate: 08/02/2016; habitat: Lowland forest disturbed by historical selective logging; **Record Level:** type: PhysicalObject; institutionID: http://grbio.org/cool/8t1f-g2z6; institutionCode: ZMJU; basisOfRecord: PreservedSpecimen
- **Type status:** Other material. **Occurrence:** individualCount: 1; sex: male; lifeStage: adult; **Taxon:** scientificName: Daphaenisca inexpectata (Durante & Zangrilli, 2016); **Location:** continent: Africa; country: Cameroon; stateProvince: Southwest Region; locality: Drink Gari, Mount Cameroon; verbatimElevation: 650 m; decimalLatitude: 04.1022; decimalLongitude: 09.0630; **Identification:** identifiedBy: Łukasz Przybyłowicz; dateIdentified: 2017; **Event:** samplingProtocol: Light catching; eventDate: 28/11/2014; habitat: Primary lowland forest; **Record Level:** type: PhysicalObject; institutionID: http://grbio.org/cool/8t1f-g2z6; institutionCode: ZMJU; basisOfRecord: PreservedSpecimen
- **Type status:** Other material. **Occurrence:** individualCount: 6; sex: males; lifeStage: adult; **Taxon:** scientificName: Daphaenisca inexpectata (Durante & Zangrilli, 2016); **Location:** continent: Africa; country: Cameroon; stateProvince: Southwest Region; locality: Drink Gari, Mount Cameroon; verbatimElevation: 650 m; decimalLatitude: 04.1022; decimalLongitude: 09.0630; **Identification:** identifiedBy: Łukasz Przybyłowicz; dateIdentified: 2017; **Event:** samplingProtocol: Light catching; eventDate: 15/04/2015; habitat: Primary lowland forest; **Record Level:** type: PhysicalObject; institutionID: http://grbio.org/cool/8t1f-g2z6; institutionCode: ZMJU; basisOfRecord: PreservedSpecimen
- **Type status:** Other material. **Occurrence:** individualCount: 2; sex: males; lifeStage: adult; **Taxon:** scientificName: Daphaenisca inexpectata (Durante & Zangrilli, 2016); **Location:** continent: Africa; country: Cameroon; stateProvince: Southwest Region; locality: Drink Gari, Mount Cameroon; verbatimElevation: 650 m; decimalLatitude: 04.1022; decimalLongitude: 09.0630; **Identification:** identifiedBy: Łukasz Przybyłowicz; dateIdentified: 2017; **Event:** samplingProtocol: Light catching; eventDate: 09/02/2016; habitat: Primary lowland forest; **Record Level:** type: PhysicalObject; institutionID: http://grbio.org/cool/8t1f-g2z6; institutionCode: ZMJU; basisOfRecord: PreservedSpecimen

#### Distribution

**Figure 7.**
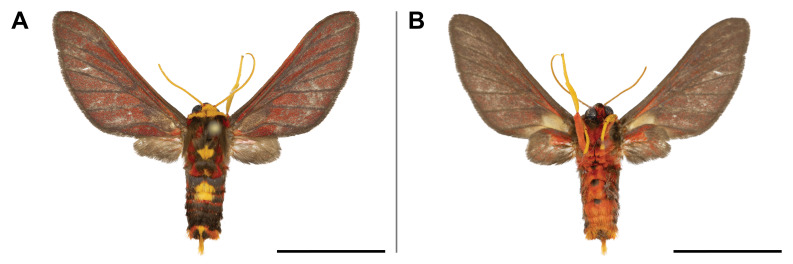
*Daphaenisca inexpectata* (Durante & Zangrilli, 2016). **A.** dorsal view; **B.** ventral view. The scale bar represents 1 cm.

This species was recently described from Gabon and considered as endemic to the country. Our record extended its known distribution to Cameroon, as well as to the Guinean biogeographic region. On Mount Cameroon, our records came from lowland forests only (350 and 650 m a.s.l.) (Fig. 7).

#### Notes

The species was originally described as Balacra (Daphaenisca) inexpectata Durante & Zangrilli, 2016. The subgenus was formally raised to genus in Przybyłowicz et al. (2019a), based on the molecular and morphological evidence. In De Prins and De Prins (2019), the junior synonym has still been listed.

### Hippurarctia judith

Kiriakoff, 1959

2D3A8AB1-E037-59EB-A23D-E941A3A598BA

Erebidae , Arctiinae

#### Materials

- **Type status:** Other material. **Occurrence:** individualCount: 7; sex: males; lifeStage: adult; **Taxon:** scientificName: Hippurarctia judith Kiriakoff, 1959; **Location:** continent: Africa; country: Cameroon; stateProvince: Southwest Region; locality: Drink Gari, Mount Cameroon; verbatimElevation: 650 m; decimalLatitude: 04.1022; decimalLongitude: 09.0630; **Identification:** identifiedBy: Łukasz Przybyłowicz; dateIdentified: 2017; **Event:** samplingProtocol: Light catching; eventDate: 05/02/2016; habitat: Primary lowland forest; **Record Level:** type: PhysicalObject; institutionID: http://grbio.org/cool/8t1f-g2z6; institutionCode: ZMJU; basisOfRecord: PreservedSpecimen
- **Type status:** Other material. **Occurrence:** individualCount: 8; sex: males; lifeStage: adult; **Taxon:** scientificName: Hippurarctia judith Kiriakoff, 1959; **Location:** continent: Africa; country: Cameroon; stateProvince: Southwest Region; locality: PlanteCam Camp, Mount Cameroon; verbatimElevation: 1,100 m; decimalLatitude: 04.1175; decimalLongitude: 09.0709; **Identification:** identifiedBy: Łukasz Przybyłowicz; dateIdentified: 2017; **Event:** samplingProtocol: Light catching; eventDate: 12/04/2015; habitat: Upland forest locally disturbed by elephants; **Record Level:** type: PhysicalObject; institutionID: http://grbio.org/cool/8t1f-g2z6; institutionCode: ZMJU; basisOfRecord: PreservedSpecimen
- **Type status:** Other material. **Occurrence:** individualCount: 24; sex: males; lifeStage: adult; **Taxon:** scientificName: Hippurarctia judith Kiriakoff, 1959; **Location:** continent: Africa; country: Cameroon; stateProvince: Southwest Region; locality: PlanteCam Camp, Mount Cameroon; verbatimElevation: 1,100 m; decimalLatitude: 04.1175; decimalLongitude: 09.0709; **Identification:** identifiedBy: Łukasz Przybyłowicz; dateIdentified: 2017; **Event:** samplingProtocol: Light catching; eventDate: 01/02/2016; habitat: Upland forest locally disturbed by elephants; **Record Level:** type: PhysicalObject; institutionID: http://grbio.org/cool/8t1f-g2z6; institutionCode: ZMJU; basisOfRecord: PreservedSpecimen
- **Type status:** Other material. **Occurrence:** individualCount: 2; sex: males; lifeStage: adult; **Taxon:** scientificName: Hippurarctia judith Kiriakoff, 1959; **Location:** continent: Africa; country: Cameroon; stateProvince: Southwest Region; locality: Crater Lake, Mount Cameroon; verbatimElevation: 1,450 m; decimalLatitude: 04.1443; decimalLongitude: 09.0717; **Identification:** identifiedBy: Łukasz Przybyłowicz; dateIdentified: 2017; **Event:** samplingProtocol: Light catching; eventDate: 20/02/2017; habitat: Submontane forest locally disturbed by elephants; **Record Level:** type: PhysicalObject; institutionID: http://grbio.org/cool/8t1f-g2z6; institutionCode: ZMJU; basisOfRecord: PreservedSpecimen

#### Distribution

**Figure 8.**
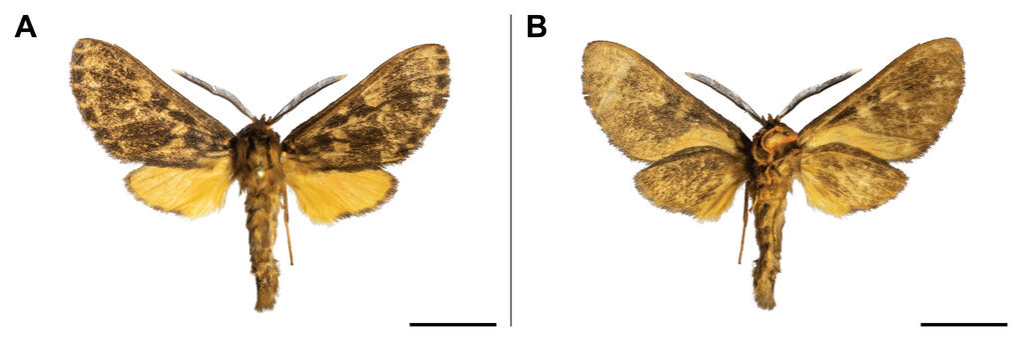
*Hippurarctia judith* Kiriakoff, 1959. **A.** dorsal view; **B.** ventral view. The scale bar represents 1 cm.

This species was known from Ghana and Democratic Republic of Congo (Przybyłowicz and Bąkowski 2011). Our record confirmed its probable occurrence across the Afrotropical rainforest zone by partially filling the distributional gap. On Mount Cameroon, it was recorded from lowland to submontane forests (650 and 1,450 m a.s.l.) (Fig. 8).

### Ligulosia costimaculata

(Aurivillius, 1910)

759F5ED5-8830-5FB7-B025-5BBAFA9E229F

Erebidae , Arctiinae

#### Materials

- **Type status:** Other material. **Occurrence:** individualCount: 1; sex: female; lifeStage: adult; **Taxon:** scientificName: Ligulosia costimaculata (Aurivillius, 1910); **Location:** continent: Africa; country: Cameroon; stateProvince: Southwest Region; locality: Drink Gari, Mount Cameroon; verbatimElevation: 650 m; decimalLatitude: 04.1022; decimalLongitude: 09.0630; **Identification:** identifiedBy: Łukasz Przybyłowicz; dateIdentified: 2017; **Event:** samplingProtocol: Light catching; eventDate: 09/02/2016; habitat: Primary lowland forest; **Record Level:** type: PhysicalObject; institutionID: http://grbio.org/cool/8t1f-g2z6; institutionCode: ZMJU; basisOfRecord: PreservedSpecimen
- **Type status:** Other material. **Occurrence:** individualCount: 55; sex: 54 males, 1 female; lifeStage: adult; **Taxon:** scientificName: Ligulosia costimaculata (Aurivillius, 1910); **Location:** continent: Africa; country: Cameroon; stateProvince: Southwest Region; locality: Crater Lake, Mount Cameroon; verbatimElevation: 1,450 m; decimalLatitude: 04.1443; decimalLongitude: 09.0717; **Identification:** identifiedBy: Łukasz Przybyłowicz; dateIdentified: 2017; **Event:** samplingProtocol: Light catching; eventDate: 24/11/2016; habitat: Submontane forest locally disturbed by elephants; **Record Level:** type: PhysicalObject; institutionID: http://grbio.org/cool/8t1f-g2z6; institutionCode: ZMJU; basisOfRecord: PreservedSpecimen
- **Type status:** Other material. **Occurrence:** individualCount: 15; sex: males; lifeStage: adult; **Taxon:** scientificName: Ligulosia costimaculata (Aurivillius, 1910); **Location:** continent: Africa; country: Cameroon; stateProvince: Southwest Region; locality: Crater Lake, Mount Cameroon; verbatimElevation: 1,450 m; decimalLatitude: 04.1443; decimalLongitude: 09.0717; **Identification:** identifiedBy: Łukasz Przybyłowicz; dateIdentified: 2017; **Event:** samplingProtocol: Light catching; eventDate: 20/02/2017; habitat: Submontane forest locally disturbed by elephants; **Record Level:** type: PhysicalObject; institutionID: http://grbio.org/cool/8t1f-g2z6; institutionCode: ZMJU; basisOfRecord: PreservedSpecimen
- **Type status:** Other material. **Occurrence:** individualCount: 15; sex: males; lifeStage: adult; **Taxon:** scientificName: Ligulosia costimaculata (Aurivillius, 1910); **Location:** continent: Africa; country: Cameroon; stateProvince: Southwest Region; locality: Crater Lake, Mount Cameroon; verbatimElevation: 1,450 m; decimalLatitude: 04.1443; decimalLongitude: 09.0717; **Identification:** identifiedBy: Łukasz Przybyłowicz; dateIdentified: 2017; **Event:** samplingProtocol: Light catching; eventDate: 26/04/2017; habitat: Submontane forest locally disturbed by elephants; **Record Level:** type: PhysicalObject; institutionID: http://grbio.org/cool/8t1f-g2z6; institutionCode: ZMJU; basisOfRecord: PreservedSpecimen
- **Type status:** Other material. **Occurrence:** individualCount: 4; sex: 3 males, 1 female; lifeStage: adult; **Taxon:** scientificName: Ligulosia costimaculata (Aurivillius, 1910); **Location:** continent: Africa; country: Cameroon; stateProvince: Southwest Region; locality: Elephant Camp, Mount Cameroon; verbatimElevation: 1,850 m; decimalLatitude: 04.1453; decimalLongitude: 09.0870; **Identification:** identifiedBy: Łukasz Przybyłowicz; dateIdentified: 2017; **Event:** samplingProtocol: Light catching; eventDate: 21/11/2014; habitat: Montane forest locally disturbed by elephants; **Record Level:** type: PhysicalObject; institutionID: http://grbio.org/cool/8t1f-g2z6; institutionCode: ZMJU; basisOfRecord: PreservedSpecimen
- **Type status:** Other material. **Occurrence:** individualCount: 32; sex: males; lifeStage: adult; **Taxon:** scientificName: Ligulosia costimaculata (Aurivillius, 1910); **Location:** continent: Africa; country: Cameroon; stateProvince: Southwest Region; locality: Elephant Camp, Mount Cameroon; verbatimElevation: 1,850 m; decimalLatitude: 04.1453; decimalLongitude: 09.0870; **Identification:** identifiedBy: Łukasz Przybyłowicz; dateIdentified: 2017; **Event:** samplingProtocol: Light catching; eventDate: 20/02/2017; habitat: Montane forest locally disturbed by elephants; **Record Level:** type: PhysicalObject; institutionID: http://grbio.org/cool/8t1f-g2z6; institutionCode: ZMJU; basisOfRecord: PreservedSpecimen
- **Type status:** Other material. **Occurrence:** individualCount: 83; sex: males; lifeStage: adult; **Taxon:** scientificName: Ligulosia costimaculata (Aurivillius, 1910); **Location:** continent: Africa; country: Cameroon; stateProvince: Southwest Region; locality: Elephant Camp, Mount Cameroon; verbatimElevation: 1,850 m; decimalLatitude: 04.1453; decimalLongitude: 09.0870; **Identification:** identifiedBy: Łukasz Przybyłowicz; dateIdentified: 2017; **Event:** samplingProtocol: Light catching; eventDate: 23/04/2017; habitat: Montane forest locally disturbed by elephants; **Record Level:** type: PhysicalObject; institutionID: http://grbio.org/cool/8t1f-g2z6; institutionCode: ZMJU; basisOfRecord: PreservedSpecimen
- **Type status:** Other material. **Occurrence:** individualCount: 9; sex: females; lifeStage: adult; **Taxon:** scientificName: Ligulosia costimaculata (Aurivillius, 1910); **Location:** continent: Africa; country: Cameroon; stateProvince: Southwest Region; locality: Mann’s Spring, Mount Cameroon; verbatimElevation: 2,200 m; decimalLatitude: 04.1428; decimalLongitude: 09.1225; **Identification:** identifiedBy: Łukasz Przybyłowicz; dateIdentified: 2017; **Event:** samplingProtocol: Light catching; eventDate: 08/11/2016; habitat: Montane forest close to the timberline; **Record Level:** type: PhysicalObject; institutionID: http://grbio.org/cool/8t1f-g2z6; institutionCode: ZMJU; basisOfRecord: PreservedSpecimen
- **Type status:** Other material. **Occurrence:** individualCount: 46; sex: females; lifeStage: adult; **Taxon:** scientificName: Ligulosia costimaculata (Aurivillius, 1910); **Location:** continent: Africa; country: Cameroon; stateProvince: Southwest Region; locality: Mann’s Spring, Mount Cameroon; verbatimElevation: 2,200 m; decimalLatitude: 04.1428; decimalLongitude: 09.1225; **Identification:** identifiedBy: Łukasz Przybyłowicz; dateIdentified: 2017; **Event:** samplingProtocol: Light catching; eventDate: 01/02/2017; habitat: Montane forest close to the timberline; **Record Level:** type: PhysicalObject; institutionID: http://grbio.org/cool/8t1f-g2z6; institutionCode: ZMJU; basisOfRecord: PreservedSpecimen
- **Type status:** Other material. **Occurrence:** individualCount: 64; sex: females; lifeStage: adult; **Taxon:** scientificName: Ligulosia costimaculata (Aurivillius, 1910); **Location:** continent: Africa; country: Cameroon; stateProvince: Southwest Region; locality: Mann’s Spring, Mount Cameroon; verbatimElevation: 2,200 m; decimalLatitude: 04.1428; decimalLongitude: 09.1225; **Identification:** identifiedBy: Łukasz Przybyłowicz; dateIdentified: 2017; **Event:** samplingProtocol: Light catching; eventDate: 18/04/2017; habitat: Montane forest close to the timberline; **Record Level:** type: PhysicalObject; institutionID: http://grbio.org/cool/8t1f-g2z6; institutionCode: ZMJU; basisOfRecord: PreservedSpecimen

#### Distribution

**Figure 9.**
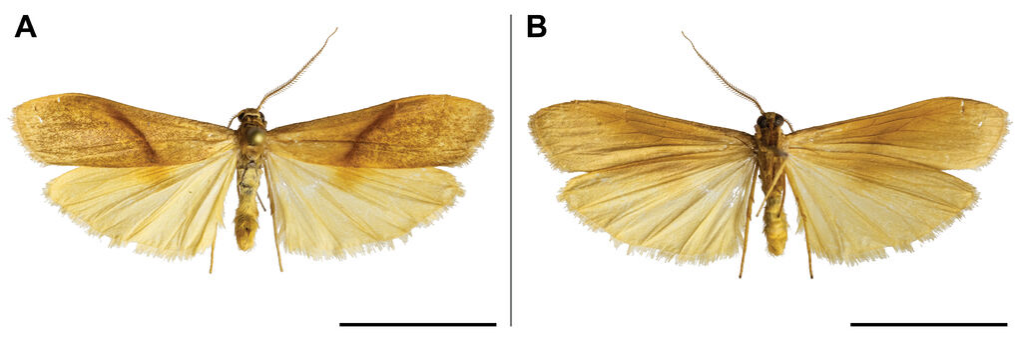
*Ligulosia costimaculata* (Aurivillius, 1910). **A.** dorsal view; **B.** ventral view. The scale bar represents 1 cm.

This species was reported from montane regions of Central (Democratic Republic of Congo) and Eastern (Kenya and Tanzania) Africa. Our record thus extended its known range to the Guinean biogeographic region. It was also the first record of *Ligulosia* genus in Cameroon. On Mount Cameroon, it has a relatively broad elevational distribution range (650–2,200 m a.s.l.), although most specimens were recorded at the higher elevations (Fig. 9).

### Palaeugoa spurrelli

(Hampson, 1914)

19304147-FFAD-5EDE-8F56-0A6C9FB74DF2

Erebidae , Arctiinae

#### Materials

- **Type status:** Other material. **Occurrence:** individualCount: 10; sex: 3 males, 7 females; lifeStage: adult; **Taxon:** scientificName: Palaeugoa spurrelli (Hampson, 1914); **Location:** continent: Africa; country: Cameroon; stateProvince: Southwest Region; locality: Bamboo Camp, Mount Cameroon; verbatimElevation: 350 m; decimalLatitude: 04.0899; decimalLongitude: 09.0517; **Identification:** identifiedBy: Łukasz Przybyłowicz; dateIdentified: 2017; **Event:** samplingProtocol: Light catching; eventDate: 09/02/2016; habitat: Lowland forest disturbed by historical selective logging; **Record Level:** type: PhysicalObject; institutionID: http://grbio.org/cool/8t1f-g2z6; institutionCode: ZMJU; basisOfRecord: PreservedSpecimen
- **Type status:** Other material. **Occurrence:** individualCount: 28; sex: 16 males, 12 females; lifeStage: adult; **Taxon:** scientificName: Palaeugoa spurrelli (Hampson, 1914); **Location:** continent: Africa; country: Cameroon; stateProvince: Southwest Region; locality: Bamboo Camp, Mount Cameroon; verbatimElevation: 350 m; decimalLatitude: 04.0899; decimalLongitude: 09.0517; **Identification:** identifiedBy: Łukasz Przybyłowicz; dateIdentified: 2017; **Event:** samplingProtocol: Light catching; eventDate: 20/04/2015; habitat: Lowland forest disturbed by historical selective logging; **Record Level:** type: PhysicalObject; institutionID: http://grbio.org/cool/8t1f-g2z6; institutionCode: ZMJU; basisOfRecord: PreservedSpecimen
- **Type status:** Other material. **Occurrence:** individualCount: 4; sex: 3 males, 1 female; lifeStage: adult; **Taxon:** scientificName: Palaeugoa spurrelli (Hampson, 1914); **Location:** continent: Africa; country: Cameroon; stateProvince: Southwest Region; locality: Bamboo Camp, Mount Cameroon; verbatimElevation: 350 m; decimalLatitude: 04.0899; decimalLongitude: 09.0517; **Identification:** identifiedBy: Łukasz Przybyłowicz; dateIdentified: 2017; **Event:** samplingProtocol: Light catching; eventDate: 17/12/2014; habitat: Lowland forest disturbed by historical selective logging; **Record Level:** type: PhysicalObject; institutionID: http://grbio.org/cool/8t1f-g2z6; institutionCode: ZMJU; basisOfRecord: PreservedSpecimen
- **Type status:** Other material. **Occurrence:** individualCount: 71; sex: 33 males, 38 females; lifeStage: adult; **Taxon:** scientificName: Palaeugoa spurrelli (Hampson, 1914); **Location:** continent: Africa; country: Cameroon; stateProvince: Southwest Region; locality: Drink Gari, Mount Cameroon; verbatimElevation: 650 m; decimalLatitude: 04.1022; decimalLongitude: 09.0630; **Identification:** identifiedBy: Łukasz Przybyłowicz; dateIdentified: 2017; **Event:** samplingProtocol: Light catching; eventDate: 04/02/2016; habitat: Primary lowland forest; **Record Level:** type: PhysicalObject; institutionID: http://grbio.org/cool/8t1f-g2z6; institutionCode: ZMJU; basisOfRecord: PreservedSpecimen
- **Type status:** Other material. **Occurrence:** individualCount: 14; sex: 12 males, 2 females; lifeStage: adult; **Taxon:** scientificName: Palaeugoa spurrelli (Hampson, 1914); **Location:** continent: Africa; country: Cameroon; stateProvince: Southwest Region; locality: Drink Gari, Mount Cameroon; verbatimElevation: 650 m; decimalLatitude: 04.1022; decimalLongitude: 09.0630; **Identification:** identifiedBy: Łukasz Przybyłowicz; dateIdentified: 2017; **Event:** samplingProtocol: Light catching; eventDate: 20/04/2015; habitat: Primary lowland forest; **Record Level:** type: PhysicalObject; institutionID: http://grbio.org/cool/8t1f-g2z6; institutionCode: ZMJU; basisOfRecord: PreservedSpecimen
- **Type status:** Other material. **Occurrence:** individualCount: 10; sex: 9 males, 1 female; lifeStage: adult; **Taxon:** scientificName: Palaeugoa spurrelli (Hampson, 1914); **Location:** continent: Africa; country: Cameroon; stateProvince: Southwest Region; locality: Drink Gari, Mount Cameroon; verbatimElevation: 650 m; decimalLatitude: 04.1022; decimalLongitude: 09.0630; **Identification:** identifiedBy: Łukasz Przybyłowicz; dateIdentified: 2017; **Event:** samplingProtocol: Light catching; eventDate: 06/12/2014; habitat: Primary lowland forest; **Record Level:** type: PhysicalObject; institutionID: http://grbio.org/cool/8t1f-g2z6; institutionCode: ZMJU; basisOfRecord: PreservedSpecimen
- **Type status:** Other material. **Occurrence:** individualCount: 5; sex: 3 males, 2 females; lifeStage: adult; **Taxon:** scientificName: Palaeugoa spurrelli (Hampson, 1914); **Location:** continent: Africa; country: Cameroon; stateProvince: Southwest Region; locality: PlanteCam Camp, Mount Cameroon; verbatimElevation: 1,100 m; decimalLatitude: 04.1175; decimalLongitude: 09.0709; **Identification:** identifiedBy: Łukasz Przybyłowicz; dateIdentified: 2017; **Event:** samplingProtocol: Light catching; eventDate: 30/01/2015; habitat: Upland forest locally disturbed by elephants; **Record Level:** type: PhysicalObject; institutionID: http://grbio.org/cool/8t1f-g2z6; institutionCode: ZMJU; basisOfRecord: PreservedSpecimen
- **Type status:** Other material. **Occurrence:** individualCount: 2; sex: females; lifeStage: adult; **Taxon:** scientificName: Palaeugoa spurrelli (Hampson, 1914); **Location:** continent: Africa; country: Cameroon; stateProvince: Southwest Region; locality: PlanteCam Camp, Mount Cameroon; verbatimElevation: 1,100 m; decimalLatitude: 04.1175; decimalLongitude: 09.0709; **Identification:** identifiedBy: Łukasz Przybyłowicz; dateIdentified: 2017; **Event:** samplingProtocol: Light catching; eventDate: 18/12/2014; habitat: Upland forest locally disturbed by elephants; **Record Level:** type: PhysicalObject; institutionID: http://grbio.org/cool/8t1f-g2z6; institutionCode: ZMJU; basisOfRecord: PreservedSpecimen

#### Distribution

**Figure 10.**
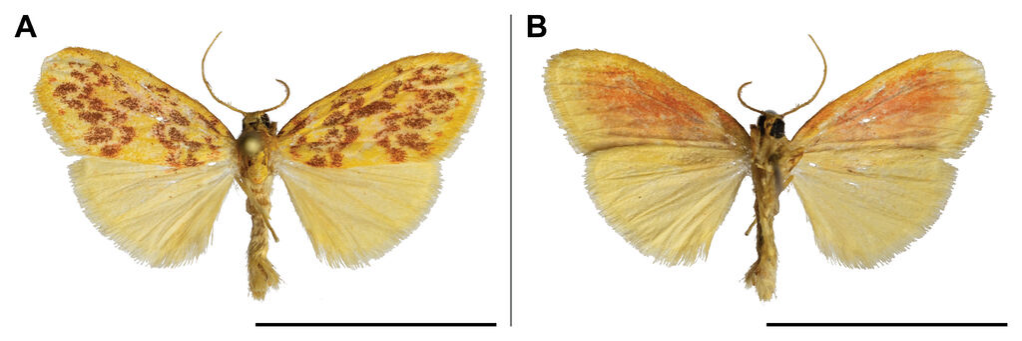
*Palaeugoa spurrelli* (Hampson, 1914). **A.** dorsal view; **B.** ventral view. The scale bar represents 1 cm.

*P. spurrelli* was reported only from Ghana and Kenya. Such scattered known distribution implies poor knowledge of the distributional range of the species. This is also the first record of the *Palaeugoa* genus in Cameroon. The two records from Mount Cameroon were made in lowland and upland forests (from 350 to 1,100 m a.s.l.) (Fig. 10).

### Calligraphidia opulenta

(Möschler, 1887)

6DF2E8BC-A03B-5865-85E4-9772267A67A1

Erebidae , Calpinae

#### Materials

- **Type status:** Other material. **Occurrence:** individualCount: 1; sex: male; lifeStage: adult; **Taxon:** scientificName: Calligraphidia opulenta (Möschler, 1887); **Location:** continent: Africa; country: Cameroon; stateProvince: Southwest Region; locality: Mapanja Camp, Mount Cameroon; verbatimElevation: 1,850 m; decimalLatitude: 04.1157; decimalLongitude: 09.1315; **Identification:** identifiedBy: Sylvain Delabye; dateIdentified: 2017; **Event:** samplingProtocol: Light catching; eventDate: 10/05/2017; habitat: Montane forest; **Record Level:** type: PhysicalObject; institutionID: http://grbio.org/cool/8t1f-g2z6; institutionCode: ZMJU; basisOfRecord: PreservedSpecimen

#### Distribution

**Figure 11.**
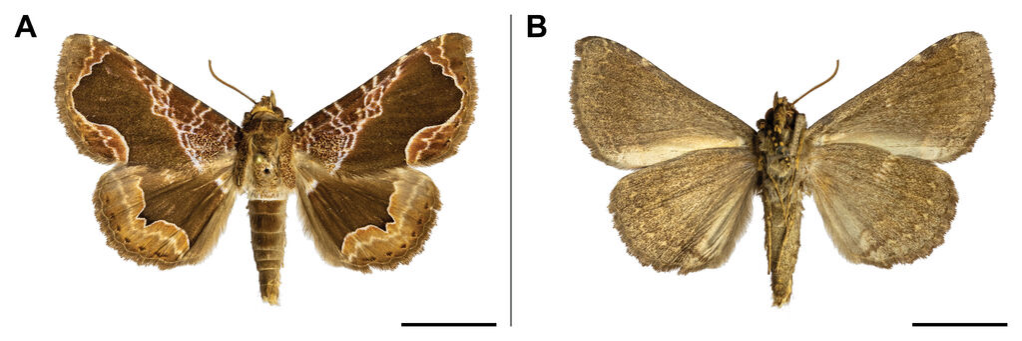
*Calligraphidia opulenta* (Möschler, 1887). **A.** dorsal view; **B.** ventral view. The scale bar represents 1 cm.

This species was known from Gabon and Ghana only. This is also the first record of the *Calligraphidia* genus in Cameroon. The only specimen from Mount Cameroon was collected in montane forest (1,850 m a.s.l.) (Fig. 11).

### Uripao albizonata

Hampson, 1926

DE9DA72C-71C8-56BB-B342-FC9B3BE3BE60

Erebidae , Calpinae

#### Materials

- **Type status:** Other material. **Occurrence:** individualCount: 1; sex: male; lifeStage: adult; **Taxon:** scientificName: Uripao albizonata Hampson, 1926; **Location:** continent: Africa; country: Cameroon; stateProvince: Southwest Region; locality: Mapanja Camp, Mount Cameroon; verbatimElevation: 1,850 m; decimalLatitude: 04.1157; decimalLongitude: 09.1315; **Identification:** identifiedBy: Sylvain Delabye; dateIdentified: 2017; **Event:** samplingProtocol: Light catching; eventDate: 15/05/2017; habitat: Montane forest; **Record Level:** type: PhysicalObject; institutionID: http://grbio.org/cool/8t1f-g2z6; institutionCode: ZMJU; basisOfRecord: PreservedSpecimen

#### Distribution

**Figure 12.**
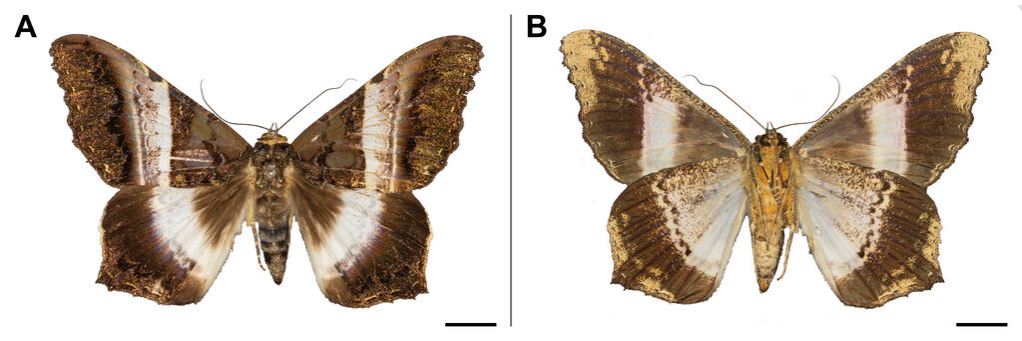
*Uripao albizonata* Hampson, 1926. **A.** dorsal view; **B.** ventral view. The scale bar represents 1 cm.

This species was known from Gabon and Sierra Leone only. This record confirmed its broader distribution. This is also the first record of the *Uripao* genus in Cameroon. On Mount Cameroon, our only record came from the montane forests (1,850 m a.s.l.) (Fig. 12).

### Dasychira punctifera

(Walker, 1858)

20A46676-D514-5C15-A48E-5B5503620587

Erebidae , Lymantriinae

#### Materials

- **Type status:** Other material. **Occurrence:** individualCount: 1; sex: male; lifeStage: adult; **Taxon:** scientificName: Dasychira punctifera (Walker, 1858); **Location:** continent: Africa; country: Cameroon; stateProvince: Southwest Region; locality: Dikolo Peninsula, Bimbia-Bonadikombo Community Forest; verbatimElevation: 30 m; decimalLatitude: 03.9818; decimalLongitude: 09.2625; **Identification:** identifiedBy: Sylvain Delabye; dateIdentified: 2017; **Event:** samplingProtocol: Light catching; eventDate: 11/10/2017; habitat: Coastal forest; **Record Level:** type: PhysicalObject; institutionID: http://grbio.org/cool/8t1f-g2z6; institutionCode: ZMJU; basisOfRecord: PreservedSpecimen
- **Type status:** Other material. **Occurrence:** individualCount: 1; sex: male; lifeStage: adult; **Taxon:** scientificName: Dasychira punctifera (Walker, 1858); **Location:** continent: Africa; country: Cameroon; stateProvince: Southwest Region; locality: Bamboo Camp, Mount Cameroon; verbatimElevation: 350 m; decimalLatitude: 04.0899; decimalLongitude: 09.0517; **Identification:** identifiedBy: Sylvain Delabye; dateIdentified: 2017; **Event:** samplingProtocol: Light catching; eventDate: 13/12/2014; habitat: Lowland forest disturbed by historical selective logging; **Record Level:** type: PhysicalObject; institutionID: http://grbio.org/cool/8t1f-g2z6; institutionCode: ZMJU; basisOfRecord: PreservedSpecimen
- **Type status:** Other material. **Occurrence:** individualCount: 9; sex: 8 males, 1 female; lifeStage: adult; **Taxon:** scientificName: Dasychira punctifera (Walker, 1858); **Location:** continent: Africa; country: Cameroon; stateProvince: Southwest Region; locality: Bamboo Camp, Mount Cameroon; verbatimElevation: 350 m; decimalLatitude: 04.0899; decimalLongitude: 09.0517; **Identification:** identifiedBy: Sylvain Delabye; dateIdentified: 2017; **Event:** samplingProtocol: Light catching; eventDate: 20/04/2015; habitat: Lowland forest disturbed by historical selective logging; **Record Level:** type: PhysicalObject; institutionID: http://grbio.org/cool/8t1f-g2z6; institutionCode: ZMJU; basisOfRecord: PreservedSpecimen
- **Type status:** Other material. **Occurrence:** individualCount: 9; sex: males; lifeStage: adult; **Taxon:** scientificName: Dasychira punctifera (Walker, 1858); **Location:** continent: Africa; country: Cameroon; stateProvince: Southwest Region; locality: Bamboo Camp, Mount Cameroon; verbatimElevation: 350 m; decimalLatitude: 04.0899; decimalLongitude: 09.0517; **Identification:** identifiedBy: Sylvain Delabye; dateIdentified: 2017; **Event:** samplingProtocol: Light catching; eventDate: 10/02/2016; habitat: Lowland forest disturbed by historical selective logging; **Record Level:** type: PhysicalObject; institutionID: http://grbio.org/cool/8t1f-g2z6; institutionCode: ZMJU; basisOfRecord: PreservedSpecimen
- **Type status:** Other material. **Occurrence:** individualCount: 1; sex: male; lifeStage: adult; **Taxon:** scientificName: Dasychira punctifera (Walker, 1858); **Location:** continent: Africa; country: Cameroon; stateProvince: Southwest Region; locality: Drink Gari, Mount Cameroon; verbatimElevation: 650 m; decimalLatitude: 04.1022; decimalLongitude: 09.0630; **Identification:** identifiedBy: Sylvain Delabye; dateIdentified: 2017; **Event:** samplingProtocol: Light catching; eventDate: 17/12/2014; habitat: Primary lowland forest; **Record Level:** type: PhysicalObject; institutionID: http://grbio.org/cool/8t1f-g2z6; institutionCode: ZMJU; basisOfRecord: PreservedSpecimen
- **Type status:** Other material. **Occurrence:** individualCount: 3; sex: males; lifeStage: adult; **Taxon:** scientificName: Dasychira punctifera (Walker, 1858); **Location:** continent: Africa; country: Cameroon; stateProvince: Southwest Region; locality: Drink Gari, Mount Cameroon; verbatimElevation: 650 m; decimalLatitude: 04.1022; decimalLongitude: 09.0630; **Identification:** identifiedBy: Sylvain Delabye; dateIdentified: 2017; **Event:** samplingProtocol: Light catching; eventDate: 22/04/2015; habitat: Primary lowland forest; **Record Level:** type: PhysicalObject; institutionID: http://grbio.org/cool/8t1f-g2z6; institutionCode: ZMJU; basisOfRecord: PreservedSpecimen
- **Type status:** Other material. **Occurrence:** individualCount: 9; sex: males; lifeStage: adult; **Taxon:** scientificName: Dasychira punctifera (Walker, 1858); **Location:** continent: Africa; country: Cameroon; stateProvince: Southwest Region; locality: Drink Gari, Mount Cameroon; verbatimElevation: 650 m; decimalLatitude: 04.1022; decimalLongitude: 09.0630; **Identification:** identifiedBy: Sylvain Delabye; dateIdentified: 2017; **Event:** samplingProtocol: Light catching; eventDate: 05/02/2016; habitat: Primary lowland forest; **Record Level:** type: PhysicalObject; institutionID: http://grbio.org/cool/8t1f-g2z6; institutionCode: ZMJU; basisOfRecord: PreservedSpecimen
- **Type status:** Other material. **Occurrence:** individualCount: 1; sex: male; lifeStage: adult; **Taxon:** scientificName: Dasychira punctifera (Walker, 1858); **Location:** continent: Africa; country: Cameroon; stateProvince: Southwest Region; locality: PlanteCam Camp, Mount Cameroon; verbatimElevation: 1,100 m; decimalLatitude: 04.1175; decimalLongitude: 09.0709; **Identification:** identifiedBy: Sylvain Delabye; dateIdentified: 2017; **Event:** samplingProtocol: Light catching; eventDate: 17/12/2014; habitat: Upland forest locally disturbed by elephants; **Record Level:** type: PhysicalObject; institutionID: http://grbio.org/cool/8t1f-g2z6; institutionCode: ZMJU; basisOfRecord: PreservedSpecimen
- **Type status:** Other material. **Occurrence:** individualCount: 4; sex: males; lifeStage: adult; **Taxon:** scientificName: Dasychira punctifera (Walker, 1858); **Location:** continent: Africa; country: Cameroon; stateProvince: Southwest Region; locality: PlanteCam Camp, Mount Cameroon; verbatimElevation: 1,100 m; decimalLatitude: 04.1175; decimalLongitude: 09.0709; **Identification:** identifiedBy: Sylvain Delabye; dateIdentified: 2017; **Event:** samplingProtocol: Light catching; eventDate: 07/04/2015; habitat: Upland forest locally disturbed by elephants; **Record Level:** type: PhysicalObject; institutionID: http://grbio.org/cool/8t1f-g2z6; institutionCode: ZMJU; basisOfRecord: PreservedSpecimen
- **Type status:** Other material. **Occurrence:** individualCount: 1; sex: male; lifeStage: adult; **Taxon:** scientificName: Dasychira punctifera (Walker, 1858); **Location:** continent: Africa; country: Cameroon; stateProvince: Southwest Region; locality: PlanteCam Camp, Mount Cameroon; verbatimElevation: 1,100 m; decimalLatitude: 04.1175; decimalLongitude: 09.0709; **Identification:** identifiedBy: Sylvain Delabye; dateIdentified: 2017; **Event:** samplingProtocol: Light catching; eventDate: 31/01/2016; habitat: Upland forest locally disturbed by elephants; **Record Level:** type: PhysicalObject; institutionID: http://grbio.org/cool/8t1f-g2z6; institutionCode: ZMJU; basisOfRecord: PreservedSpecimen
- **Type status:** Other material. **Occurrence:** individualCount: 1; sex: male; lifeStage: adult; **Taxon:** scientificName: Dasychira punctifera (Walker, 1858); **Location:** continent: Africa; country: Cameroon; stateProvince: Southwest Region; locality: Crater Lake, Mount Cameroon; verbatimElevation: 1,450 m; decimalLatitude: 04.1443; decimalLongitude: 09.0717; **Identification:** identifiedBy: Sylvain Delabye; dateIdentified: 2017; **Event:** samplingProtocol: Light catching; eventDate: 28/11/2016; habitat: Submontane forest locally disturbed by elephants; **Record Level:** type: PhysicalObject; institutionID: http://grbio.org/cool/8t1f-g2z6; institutionCode: ZMJU; basisOfRecord: PreservedSpecimen
- **Type status:** Other material. **Occurrence:** individualCount: 1; sex: male; lifeStage: adult; **Taxon:** scientificName: Dasychira punctifera (Walker, 1858); **Location:** continent: Africa; country: Cameroon; stateProvince: Southwest Region; locality: Crater Lake, Mount Cameroon; verbatimElevation: 1,450 m; decimalLatitude: 04.1443; decimalLongitude: 09.0717; **Identification:** identifiedBy: Sylvain Delabye; dateIdentified: 2017; **Event:** samplingProtocol: Light catching; eventDate: 19/02/2017; habitat: Submontane forest locally disturbed by elephants; **Record Level:** type: PhysicalObject; institutionID: http://grbio.org/cool/8t1f-g2z6; institutionCode: ZMJU; basisOfRecord: PreservedSpecimen
- **Type status:** Other material. **Occurrence:** individualCount: 1; sex: male; lifeStage: adult; **Taxon:** scientificName: Dasychira punctifera (Walker, 1858); **Location:** continent: Africa; country: Cameroon; stateProvince: Southwest Region; locality: Elephant Camp, Mount Cameroon; verbatimElevation: 1,850 m; decimalLatitude: 04.1453; decimalLongitude: 09.0870; **Identification:** identifiedBy: Sylvain Delabye; dateIdentified: 2017; **Event:** samplingProtocol: Light catching; eventDate: 19/02/2017; habitat: Montane forest locally disturbed by elephants; **Record Level:** type: PhysicalObject; institutionID: http://grbio.org/cool/8t1f-g2z6; institutionCode: ZMJU; basisOfRecord: PreservedSpecimen

#### Distribution

**Figure 13.**
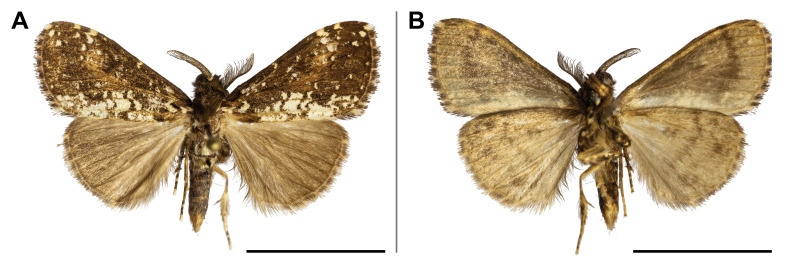
*Dasychira punctifera* (Walker, 1858). **A.** dorsal view; **B.** ventral view. The scale bar represents 1 cm.

*D. punctifera* was known from several countries across Central and Eastern Africa (but none bordering with Cameroon), but also in Côte d’Ivoire and South Africa. In the Mount Cameroon region, it has a very broad elevational range from 30 m to 1,850 m a.s.l. (Fig. 13).

### Euproctis ceramozona

Collenette, 1931

0C0EABA1-3D0B-546A-BA0E-3EC39A0D5DA0

Erebidae , Lymantriinae

#### Materials

- **Type status:** Other material. **Occurrence:** individualCount: 1; sex: female; lifeStage: adult; **Taxon:** scientificName: Euproctis ceramozona Collenette, 1931; **Location:** continent: Africa; country: Cameroon; stateProvince: Southwest Region; locality: Dikolo Peninsula, Bimbia-Bonadikombo Community Forest; verbatimElevation: 30 m; decimalLatitude: 03.9818; decimalLongitude: 09.2625; **Identification:** identifiedBy: Sylvain Delabye; dateIdentified: 2017; **Event:** samplingProtocol: Light catching; eventDate: 15/01/2016; habitat: Coastal forest; **Record Level:** type: PhysicalObject; institutionID: http://grbio.org/cool/8t1f-g2z6; institutionCode: ZMJU; basisOfRecord: PreservedSpecimen
- **Type status:** Other material. **Occurrence:** individualCount: 3; sex: males; lifeStage: adult; **Taxon:** scientificName: Euproctis ceramozona Collenette, 1931; **Location:** continent: Africa; country: Cameroon; stateProvince: Southwest Region; locality: Bamboo Camp, Mount Cameroon; verbatimElevation: 350 m; decimalLatitude: 04.0899; decimalLongitude: 09.0517; **Identification:** identifiedBy: Sylvain Delabye; dateIdentified: 2017; **Event:** samplingProtocol: Light catching; eventDate: 15/12/2014; habitat: Lowland forest disturbed by historical selective logging; **Record Level:** type: PhysicalObject; institutionID: http://grbio.org/cool/8t1f-g2z6; institutionCode: ZMJU; basisOfRecord: PreservedSpecimen
- **Type status:** Other material. **Occurrence:** individualCount: 5; sex: males; lifeStage: adult; **Taxon:** scientificName: Euproctis ceramozona Collenette, 1931; **Location:** continent: Africa; country: Cameroon; stateProvince: Southwest Region; locality: Bamboo Camp, Mount Cameroon; verbatimElevation: 350 m; decimalLatitude: 04.0899; decimalLongitude: 09.0517; **Identification:** identifiedBy: Sylvain Delabye; dateIdentified: 2017; **Event:** samplingProtocol: Light catching; eventDate: 20/04/2015; habitat: Lowland forest disturbed by historical selective logging; **Record Level:** type: PhysicalObject; institutionID: http://grbio.org/cool/8t1f-g2z6; institutionCode: ZMJU; basisOfRecord: PreservedSpecimen
- **Type status:** Other material. **Occurrence:** individualCount: 24; sex: 21 males, 3 females; lifeStage: adult; **Taxon:** scientificName: Euproctis ceramozona Collenette, 1931; **Location:** continent: Africa; country: Cameroon; stateProvince: Southwest Region; locality: Bamboo Camp, Mount Cameroon; verbatimElevation: 350 m; decimalLatitude: 04.0899; decimalLongitude: 09.0517; **Identification:** identifiedBy: Sylvain Delabye; dateIdentified: 2017; **Event:** samplingProtocol: Light catching; eventDate: 09/02/2016; habitat: Lowland forest disturbed by historical selective logging; **Record Level:** type: PhysicalObject; institutionID: http://grbio.org/cool/8t1f-g2z6; institutionCode: ZMJU; basisOfRecord: PreservedSpecimen
- **Type status:** Other material. **Occurrence:** individualCount: 2; sex: males; lifeStage: adult; **Taxon:** scientificName: Euproctis ceramozona Collenette, 1931; **Location:** continent: Africa; country: Cameroon; stateProvince: Southwest Region; locality: Drink Gari, Mount Cameroon; verbatimElevation: 650 m; decimalLatitude: 04.1022; decimalLongitude: 09.0630; **Identification:** identifiedBy: Sylvain Delabye; dateIdentified: 2017; **Event:** samplingProtocol: Light catching; eventDate: 17/04/2015; habitat: Primary lowland forest; **Record Level:** type: PhysicalObject; institutionID: http://grbio.org/cool/8t1f-g2z6; institutionCode: ZMJU; basisOfRecord: PreservedSpecimen
- **Type status:** Other material. **Occurrence:** individualCount: 12; sex: 10 males, 2 females; lifeStage: adult; **Taxon:** scientificName: Euproctis ceramozona Collenette, 1931; **Location:** continent: Africa; country: Cameroon; stateProvince: Southwest Region; locality: Drink Gari, Mount Cameroon; verbatimElevation: 650 m; decimalLatitude: 04.1022; decimalLongitude: 09.0630; **Identification:** identifiedBy: Sylvain Delabye; dateIdentified: 2017; **Event:** samplingProtocol: Light catching; eventDate: 05/02/2016; habitat: Primary lowland forest; **Record Level:** type: PhysicalObject; institutionID: http://grbio.org/cool/8t1f-g2z6; institutionCode: ZMJU; basisOfRecord: PreservedSpecimen
- **Type status:** Other material. **Occurrence:** individualCount: 1; sex: male; lifeStage: adult; **Taxon:** scientificName: Euproctis ceramozona Collenette, 1931; **Location:** continent: Africa; country: Cameroon; stateProvince: Southwest Region; locality: PlanteCam Camp, Mount Cameroon; verbatimElevation: 1,100 m; decimalLatitude: 04.1175; decimalLongitude: 09.0709; **Identification:** identifiedBy: Sylvain Delabye; dateIdentified: 2017; **Event:** samplingProtocol: Light catching; eventDate: 09/04/2015; habitat: Upland forest locally disturbed by elephants; **Record Level:** type: PhysicalObject; institutionID: http://grbio.org/cool/8t1f-g2z6; institutionCode: ZMJU; basisOfRecord: PreservedSpecimen

#### Distribution

**Figure 14.**
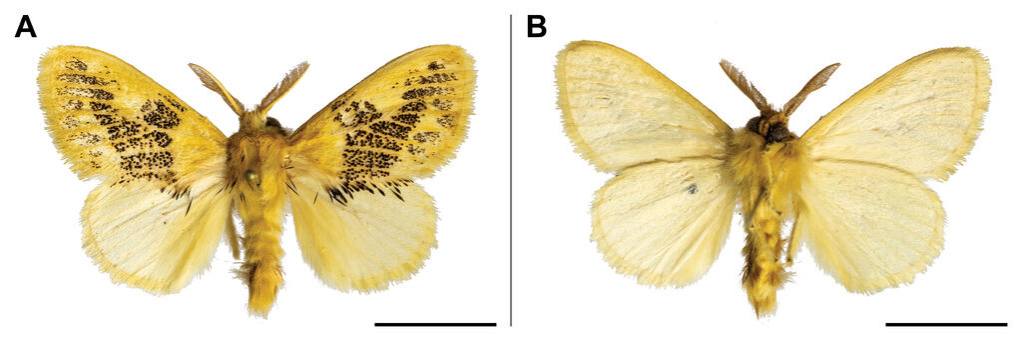
*Euproctis ceramozona* Collenette, 1931. **A.** dorsal view; **B.** ventral view. The scale bar represents 1 cm.

This species was known from Ghana and Nigeria. Our record broadened its known distribution eastwards. In the Mount Cameroon region, we recorded it in all sampled lowland and upland forests up to 1,100 m a.s.l. (Fig. 14).

### Lomadonta saturata

Swinhoe, 1904

9918C48B-09E1-55D2-97E5-EEF2E23198AB

Erebidae , Lymantriinae

#### Materials

- **Type status:** Other material. **Occurrence:** individualCount: 1; sex: male; lifeStage: adult; **Taxon:** scientificName: Lomadonta saturata Swinhoe, 1904; **Location:** continent: Africa; country: Cameroon; stateProvince: Southwest Region; locality: Bamboo Camp, Mount Cameroon; verbatimElevation: 350 m; decimalLatitude: 04.0899; decimalLongitude: 09.0517; **Identification:** identifiedBy: Sylvain Delabye; dateIdentified: 2017; **Event:** samplingProtocol: Light catching; eventDate: 22/04/2015; habitat: Lowland forest disturbed by historical selective logging; **Record Level:** type: PhysicalObject; institutionID: http://grbio.org/cool/8t1f-g2z6; institutionCode: ZMJU; basisOfRecord: PreservedSpecimen
- **Type status:** Other material. **Occurrence:** individualCount: 2; sex: males; lifeStage: adult; **Taxon:** scientificName: Lomadonta saturata Swinhoe, 1904; **Location:** continent: Africa; country: Cameroon; stateProvince: Southwest Region; locality: Bamboo Camp, Mount Cameroon; verbatimElevation: 350 m; decimalLatitude: 04.0899; decimalLongitude: 09.0517; **Identification:** identifiedBy: Sylvain Delabye; dateIdentified: 2017; **Event:** samplingProtocol: Light catching; eventDate: 10/02/2016; habitat: Lowland forest disturbed by historical selective logging; **Record Level:** type: PhysicalObject; institutionID: http://grbio.org/cool/8t1f-g2z6; institutionCode: ZMJU; basisOfRecord: PreservedSpecimen
- **Type status:** Other material. **Occurrence:** individualCount: 1; sex: male; lifeStage: adult; **Taxon:** scientificName: Lomadonta saturata Swinhoe, 1904; **Location:** continent: Africa; country: Cameroon; stateProvince: Southwest Region; locality: Drink Gari, Mount Cameroon; verbatimElevation: 650 m; decimalLatitude: 04.1022; decimalLongitude: 09.0630; **Identification:** identifiedBy: Sylvain Delabye; dateIdentified: 2017; **Event:** samplingProtocol: Light catching; eventDate: 16/04/2015; habitat: Primary lowland forest; **Record Level:** type: PhysicalObject; institutionID: http://grbio.org/cool/8t1f-g2z6; institutionCode: ZMJU; basisOfRecord: PreservedSpecimen
- **Type status:** Other material. **Occurrence:** individualCount: 5; sex: 4 males, 1 female; lifeStage: adult; **Taxon:** scientificName: Lomadonta saturata Swinhoe, 1904; **Location:** continent: Africa; country: Cameroon; stateProvince: Southwest Region; locality: Drink Gari, Mount Cameroon; verbatimElevation: 650 m; decimalLatitude: 04.1022; decimalLongitude: 09.0630; **Identification:** identifiedBy: Sylvain Delabye; dateIdentified: 2017; **Event:** samplingProtocol: Light catching; eventDate: 05/02/2016; habitat: Primary lowland forest; **Record Level:** type: PhysicalObject; institutionID: http://grbio.org/cool/8t1f-g2z6; institutionCode: ZMJU; basisOfRecord: PreservedSpecimen
- **Type status:** Other material. **Occurrence:** individualCount: 1; sex: male; lifeStage: adult; **Taxon:** scientificName: Lomadonta saturata Swinhoe, 1904; **Location:** continent: Africa; country: Cameroon; stateProvince: Southwest Region; locality: PlanteCam Camp, Mount Cameroon; verbatimElevation: 1,100 m; decimalLatitude: 04.1175; decimalLongitude: 09.0709; **Identification:** identifiedBy: Sylvain Delabye; dateIdentified: 2017; **Event:** samplingProtocol: Light catching; eventDate: 01/02/2016; habitat: Upland forest locally disturbed by elephants; **Record Level:** type: PhysicalObject; institutionID: http://grbio.org/cool/8t1f-g2z6; institutionCode: ZMJU; basisOfRecord: PreservedSpecimen
- **Type status:** Other material. **Occurrence:** individualCount: 2; sex: males; lifeStage: adult; **Taxon:** scientificName: Mimopacha tripunctata (Aurivillius, 1905); **Location:** continent: Africa; country: Cameroon; stateProvince: Southwest Region; locality: Crater Lake, Mount Cameroon; verbatimElevation: 1,450 m; decimalLatitude: 04.1443; decimalLongitude: 09.0717; **Identification:** identifiedBy: Vincent Maicher; dateIdentified: 2017; **Event:** samplingProtocol: Light catching; eventDate: 24/11/2016; habitat: Submontane forest locally disturbed by elephants; **Record Level:** type: PhysicalObject; institutionID: http://grbio.org/cool/8t1f-g2z6; institutionCode: ZMJU; basisOfRecord: PreservedSpecimen
- **Type status:** Other material. **Occurrence:** individualCount: 2; sex: males; lifeStage: adult; **Taxon:** scientificName: Mimopacha tripunctata (Aurivillius, 1905); **Location:** continent: Africa; country: Cameroon; stateProvince: Southwest Region; locality: Elephant Camp, Mount Cameroon; verbatimElevation: 1,850 m; decimalLatitude: 04.1453; decimalLongitude: 09.0870; **Identification:** identifiedBy: Vincent Maicher; dateIdentified: 2017; **Event:** samplingProtocol: Light catching; eventDate: 18/11/2014; habitat: Montane forest locally disturbed by elephants; **Record Level:** type: PhysicalObject; institutionID: http://grbio.org/cool/8t1f-g2z6; institutionCode: ZMJU; basisOfRecord: PreservedSpecimen
- **Type status:** Other material. **Occurrence:** individualCount: 2; sex: males; lifeStage: adult; **Taxon:** scientificName: Mimopacha tripunctata (Aurivillius, 1905); **Location:** continent: Africa; country: Cameroon; stateProvince: Southwest Region; locality: Mapanja Camp, Mount Cameroon; verbatimElevation: 1,850 m; decimalLatitude: 04.1157; decimalLongitude: 09.1315; **Identification:** identifiedBy: Vincent Maicher; dateIdentified: 2017; **Event:** samplingProtocol: Light catching; eventDate: 24/10/2017; habitat: Montane forest; **Record Level:** type: PhysicalObject; institutionID: http://grbio.org/cool/8t1f-g2z6; institutionCode: ZMJU; basisOfRecord: PreservedSpecimen

#### Distribution

**Figure 15.**
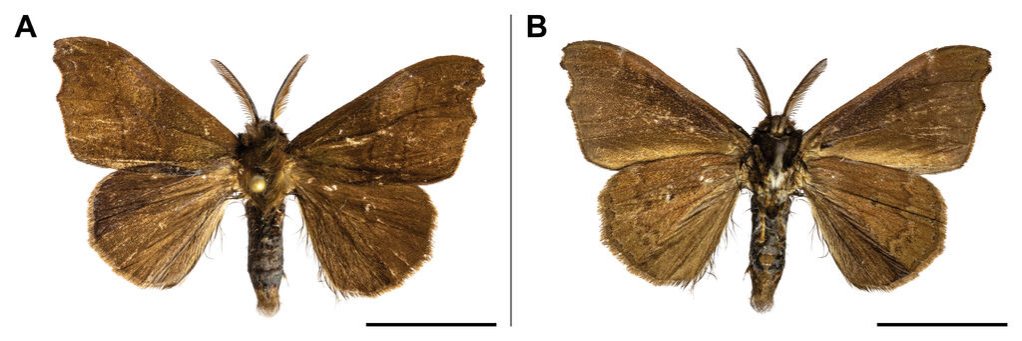
*Lomadonta saturata* Swinhoe, 1904. **A.** dorsal view; **B.** ventral view. The scale bar represents 1 cm.

This species was considered as endemic to Nigeria; we extended its known distribution eastwards. On Mount Cameroon, it was collected in forests from 350 to 1,100 m a.s.l. (Fig. 15).

### Orgyia basalis

(Walker, 1855)

7B983617-8444-54B6-9AB2-6790F0BA111E

Erebidae , Lymantriinae

#### Materials

- **Type status:** Other material. **Occurrence:** individualCount: 2; sex: males; lifeStage: adult; **Taxon:** scientificName: Orgyia basalis (Walker, 1855); **Location:** continent: Africa; country: Cameroon; stateProvince: Southwest Region; locality: Bamboo Camp, Mount Cameroon; verbatimElevation: 350 m; decimalLatitude: 04.0899; decimalLongitude: 09.0517; **Identification:** identifiedBy: Sylvain Delabye; dateIdentified: 2017; **Event:** samplingProtocol: Light catching; eventDate: 15/12/2014; habitat: Lowland forest disturbed by historical selective logging; **Record Level:** type: PhysicalObject; institutionID: http://grbio.org/cool/8t1f-g2z6; institutionCode: ZMJU; basisOfRecord: PreservedSpecimen
- **Type status:** Other material. **Occurrence:** individualCount: 3; sex: males; lifeStage: adult; **Taxon:** scientificName: Orgyia basalis (Walker, 1855); **Location:** continent: Africa; country: Cameroon; stateProvince: Southwest Region; locality: Bamboo Camp, Mount Cameroon; verbatimElevation: 350 m; decimalLatitude: 04.0899; decimalLongitude: 09.0517; **Identification:** identifiedBy: Sylvain Delabye; dateIdentified: 2017; **Event:** samplingProtocol: Light catching; eventDate: 20/04/2015; habitat: Lowland forest disturbed by historical selective logging; **Record Level:** type: PhysicalObject; institutionID: http://grbio.org/cool/8t1f-g2z6; institutionCode: ZMJU; basisOfRecord: PreservedSpecimen
- **Type status:** Other material. **Occurrence:** individualCount: 9; sex: 8 males, 1 female; lifeStage: adult; **Taxon:** scientificName: Orgyia basalis (Walker, 1855); **Location:** continent: Africa; country: Cameroon; stateProvince: Southwest Region; locality: Bamboo Camp, Mount Cameroon; verbatimElevation: 350 m; decimalLatitude: 04.0899; decimalLongitude: 09.0517; **Identification:** identifiedBy: Sylvain Delabye; dateIdentified: 2017; **Event:** samplingProtocol: Light catching; eventDate: 09/02/2016; habitat: Lowland forest disturbed by historical selective logging; **Record Level:** type: PhysicalObject; institutionID: http://grbio.org/cool/8t1f-g2z6; institutionCode: ZMJU; basisOfRecord: PreservedSpecimen
- **Type status:** Other material. **Occurrence:** individualCount: 2; sex: males; lifeStage: adult; **Taxon:** scientificName: Orgyia basalis (Walker, 1855); **Location:** continent: Africa; country: Cameroon; stateProvince: Southwest Region; locality: Drink Gari, Mount Cameroon; verbatimElevation: 650 m; decimalLatitude: 04.1022; decimalLongitude: 09.0630; **Identification:** identifiedBy: Sylvain Delabye; dateIdentified: 2017; **Event:** samplingProtocol: Light catching; eventDate: 14/11/2015; habitat: Primary lowland forest; **Record Level:** type: PhysicalObject; institutionID: http://grbio.org/cool/8t1f-g2z6; institutionCode: ZMJU; basisOfRecord: PreservedSpecimen
- **Type status:** Other material. **Occurrence:** individualCount: 5; sex: males; lifeStage: adult; **Taxon:** scientificName: Orgyia basalis (Walker, 1855); **Location:** continent: Africa; country: Cameroon; stateProvince: Southwest Region; locality: Drink Gari, Mount Cameroon; verbatimElevation: 650 m; decimalLatitude: 04.1022; decimalLongitude: 09.0630; **Identification:** identifiedBy: Sylvain Delabye; dateIdentified: 2017; **Event:** samplingProtocol: Light catching; eventDate: 04/02/2016; habitat: Primary lowland forest; **Record Level:** type: PhysicalObject; institutionID: http://grbio.org/cool/8t1f-g2z6; institutionCode: ZMJU; basisOfRecord: PreservedSpecimen
- **Type status:** Other material. **Occurrence:** individualCount: 2; sex: males; lifeStage: adult; **Taxon:** scientificName: Orgyia basalis (Walker, 1855); **Location:** continent: Africa; country: Cameroon; stateProvince: Southwest Region; locality: PlanteCam Camp, Mount Cameroon; verbatimElevation: 1,100 m; decimalLatitude: 04.1175; decimalLongitude: 09.0709; **Identification:** identifiedBy: Sylvain Delabye; dateIdentified: 2017; **Event:** samplingProtocol: Light catching; eventDate: 13/04/2015; habitat: Upland forest locally disturbed by elephants; **Record Level:** type: PhysicalObject; institutionID: http://grbio.org/cool/8t1f-g2z6; institutionCode: ZMJU; basisOfRecord: PreservedSpecimen

#### Distribution

**Figure 16.**
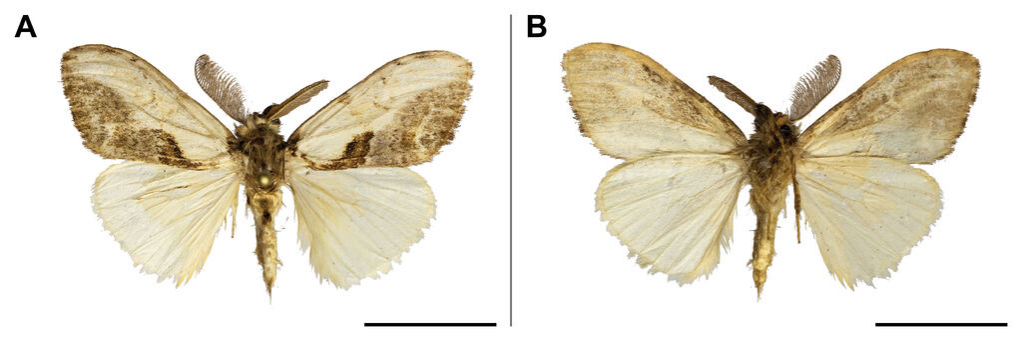
*Orgyia basalis* (Walker, 1855). **A.** dorsal view; **B.** ventral view. The scale bar represents 1 cm.

This species was previously recorded only in Sierra Leone and Zimbabwe; our record thus partly filled the wide gap in its distribution. It is also the first record of the *Orgyia* genus in Cameroon. On Mount Cameroon, it was recorded in lowland and upland forests ranges from 350 to 1,100 m a.s.l. (Fig. 16).

### Pirga ubangiana

Schultze, 1934

550761F1-ECAD-5920-B884-4AD485873420

Erebidae , Lymantriinae

#### Materials

- **Type status:** Other material. **Occurrence:** individualCount: 2; sex: females; lifeStage: adult; **Taxon:** scientificName: Pirga ubangiana Schultze, 1934; **Location:** continent: Africa; country: Cameroon; stateProvince: Southwest Region; locality: Bamboo Camp, Mount Cameroon; verbatimElevation: 350 m; decimalLatitude: 04.0899; decimalLongitude: 09.0517; **Identification:** identifiedBy: Sylvain Delabye; dateIdentified: 2017; **Event:** samplingProtocol: Light catching; eventDate: 20/04/2015; habitat: Lowland forest disturbed by historical selective logging; **Record Level:** type: PhysicalObject; institutionID: http://grbio.org/cool/8t1f-g2z6; institutionCode: ZMJU; basisOfRecord: PreservedSpecimen
- **Type status:** Other material. **Occurrence:** individualCount: 2; sex: females; lifeStage: adult; **Taxon:** scientificName: Pirga ubangiana Schultze, 1934; **Location:** continent: Africa; country: Cameroon; stateProvince: Southwest Region; locality: Drink Gari, Mount Cameroon; verbatimElevation: 650 m; decimalLatitude: 04.1022; decimalLongitude: 09.0630; **Identification:** identifiedBy: Sylvain Delabye; dateIdentified: 2017; **Event:** samplingProtocol: Light catching; eventDate: 03/02/2016; habitat: Primary lowland forest; **Record Level:** type: PhysicalObject; institutionID: http://grbio.org/cool/8t1f-g2z6; institutionCode: ZMJU; basisOfRecord: PreservedSpecimen

#### Distribution

**Figure 17.**
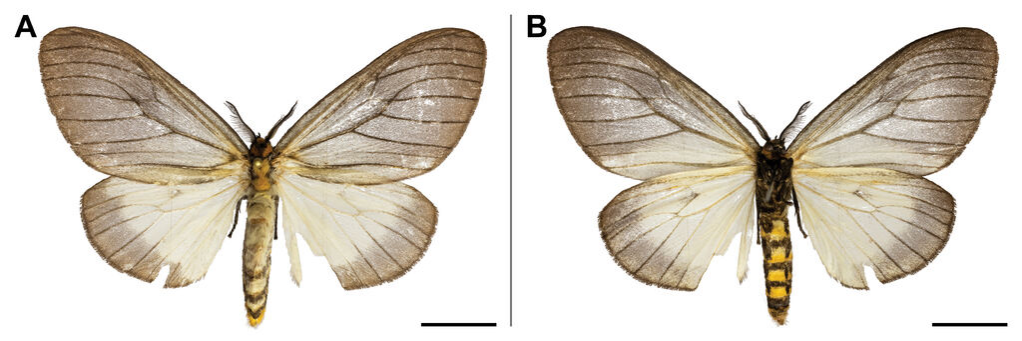
*Pirga ubangiana* Schultze, 1934. **A.** dorsal view; **B.** ventral view. The scale bar represents 1 cm.

This species was known only from Uganda and Central African Republic. Our Cameroonian record thus broadened its distribution range westwards to the Guinean biogeographic region. On Mount Cameroon, it was collected in lowland forests at 350 and 650 m a.s.l. (Fig. 17).

### Rhypopteryx rubripunctata

(Weymer, 1892)

0D3BC1E9-4AAD-58E4-B9F3-51D72433283C

Erebidae , Lymantriinae

#### Materials

- **Type status:** Other material. **Occurrence:** individualCount: 6; sex: males; lifeStage: adult; **Taxon:** scientificName: Rhypopteryx rubripunctata (Weymer, 1892); **Location:** continent: Africa; country: Cameroon; stateProvince: Southwest Region; locality: Dikolo Peninsula, Bimbia-Bonadikombo Community Forest; verbatimElevation: 30 m; decimalLatitude: 03.9818; decimalLongitude: 09.2625; **Identification:** identifiedBy: Sylvain Delabye; dateIdentified: 2017; **Event:** samplingProtocol: Light catching; eventDate: 14/01/2016; habitat: Coastal forest; **Record Level:** type: PhysicalObject; institutionID: http://grbio.org/cool/8t1f-g2z6; institutionCode: ZMJU; basisOfRecord: PreservedSpecimen
- **Type status:** Other material. **Occurrence:** individualCount: 1; sex: male; lifeStage: adult; **Taxon:** scientificName: Rhypopteryx rubripunctata (Weymer, 1892); **Location:** continent: Africa; country: Cameroon; stateProvince: Southwest Region; locality: Bamboo Camp, Mount Cameroon; verbatimElevation: 350 m; decimalLatitude: 04.0899; decimalLongitude: 09.0517; **Identification:** identifiedBy: Sylvain Delabye; dateIdentified: 2017; **Event:** samplingProtocol: Light catching; eventDate: 19/12/2014; habitat: Lowland forest disturbed by historical selective logging; **Record Level:** type: PhysicalObject; institutionID: http://grbio.org/cool/8t1f-g2z6; institutionCode: ZMJU; basisOfRecord: PreservedSpecimen
- **Type status:** Other material. **Occurrence:** individualCount: 1; sex: male; lifeStage: adult; **Taxon:** scientificName: Rhypopteryx rubripunctata (Weymer, 1892); **Location:** continent: Africa; country: Cameroon; stateProvince: Southwest Region; locality: Bamboo Camp, Mount Cameroon; verbatimElevation: 350 m; decimalLatitude: 04.0899; decimalLongitude: 09.0517; **Identification:** identifiedBy: Sylvain Delabye; dateIdentified: 2017; **Event:** samplingProtocol: Light catching; eventDate: 17/04/2015; habitat: Lowland forest disturbed by historical selective logging; **Record Level:** type: PhysicalObject; institutionID: http://grbio.org/cool/8t1f-g2z6; institutionCode: ZMJU; basisOfRecord: PreservedSpecimen
- **Type status:** Other material. **Occurrence:** individualCount: 17; sex: females; lifeStage: adult; **Taxon:** scientificName: Rhypopteryx rubripunctata (Weymer, 1892); **Location:** continent: Africa; country: Cameroon; stateProvince: Southwest Region; locality: Bamboo Camp, Mount Cameroon; verbatimElevation: 350 m; decimalLatitude: 04.0899; decimalLongitude: 09.0517; **Identification:** identifiedBy: Sylvain Delabye; dateIdentified: 2017; **Event:** samplingProtocol: Light catching; eventDate: 09/02/2016; habitat: Lowland forest disturbed by historical selective logging; **Record Level:** type: PhysicalObject; institutionID: http://grbio.org/cool/8t1f-g2z6; institutionCode: ZMJU; basisOfRecord: PreservedSpecimen
- **Type status:** Other material. **Occurrence:** individualCount: 1; sex: males; lifeStage: adult; **Taxon:** scientificName: Rhypopteryx rubripunctata (Weymer, 1892); **Location:** continent: Africa; country: Cameroon; stateProvince: Southwest Region; locality: Drink Gari, Mount Cameroon; verbatimElevation: 650 m; decimalLatitude: 04.1022; decimalLongitude: 09.0630; **Identification:** identifiedBy: Sylvain Delabye; dateIdentified: 2017; **Event:** samplingProtocol: Light catching; eventDate: 09/02/2017; habitat: Primary lowland forest; **Record Level:** type: PhysicalObject; institutionID: http://grbio.org/cool/8t1f-g2z6; institutionCode: ZMJU; basisOfRecord: PreservedSpecimen

#### Distribution

**Figure 18.**
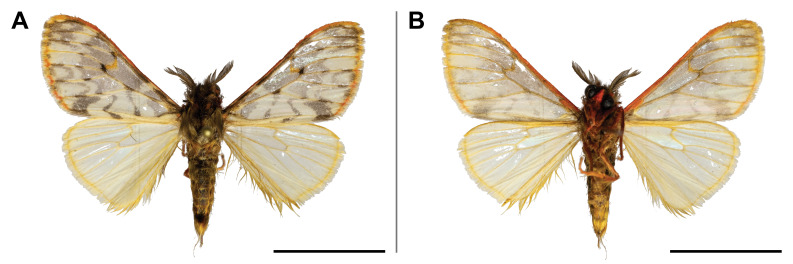
*Rhypopteryx rubripunctata* (Weymer, 1892). **A.** dorsal view; **B.** ventral view. The scale bar represents 1 cm.

This species was known from the Democratic Republic of Congo, Tanzania and South Africa. Our record in Cameroon is thus the westernmost for the species and extended its distribution to the Guinean biogeographic region. In the Mount Cameroon region, it was recorded in all studied lowland forests up to 650 m a.s.l. (Fig. 18).

### Stenoglene plagiatus

(Aurivillius, 1911)

1A905E82-28AD-5D1C-9FEF-2E0CA719819A

Eupterotidae , Janinae

#### Materials

- **Type status:** Other material. **Occurrence:** individualCount: 1; sex: male; lifeStage: adult; **Taxon:** scientificName: Stenoglene plagiatus (Aurivillius, 1911); **Location:** continent: Africa; country: Cameroon; stateProvince: Southwest Region; locality: Dikolo Peninsula, Bimbia-Bonadikombo Community Forest; verbatimElevation: 30 m; decimalLatitude: 03.9818; decimalLongitude: 09.2625; **Identification:** identifiedBy: Vincent Maicher; dateIdentified: 2017; **Event:** samplingProtocol: Light catching; eventDate: 17/01/2016; habitat: Coastal forest; **Record Level:** type: PhysicalObject; institutionID: http://grbio.org/cool/8t1f-g2z6; institutionCode: ZMJU; basisOfRecord: PreservedSpecimen

#### Distribution

**Figure 19.**
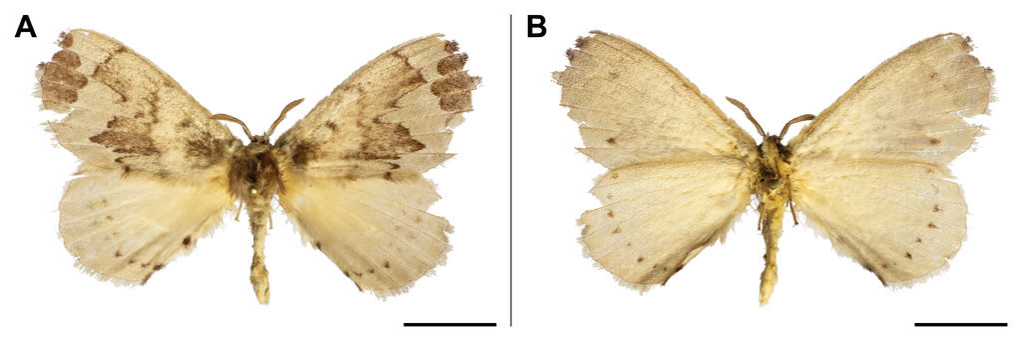
*Stenoglene plagiatus* (Aurivillius, 1911). **A.** dorsal view; **B.** ventral view. The scale bar represents 1 cm.

The known distribution of this species already included the Guinean (Ghana) and Congolian (Gabon, Central African Republic and Democratic Republic of Congo) biogeographic regions. The only specimen was collected in coastal forest at 30 m a.s.l. (Fig. 19).

### Hypotrabala castanea

Holland, 1893

A1EF05FC-F4EE-5D91-9AA5-681F7B1E19A2

Lasiocampidae , Lasiocampinae

#### Materials

- **Type status:** Other material. **Occurrence:** individualCount: 1; sex: male; lifeStage: adult; **Taxon:** scientificName: Hypotrabala castanea Holland, 1893; **Location:** continent: Africa; country: Cameroon; stateProvince: Southwest Region; locality: Dikolo Peninsula, Bimbia-Bonadikombo Community Forest; verbatimElevation: 30 m; decimalLatitude: 03.9818; decimalLongitude: 09.2625; **Identification:** identifiedBy: Vincent Maicher; dateIdentified: 2017; **Event:** samplingProtocol: Light catching; eventDate: 10/10/2017; habitat: Coastal forest; **Record Level:** type: PhysicalObject; institutionID: http://grbio.org/cool/8t1f-g2z6; institutionCode: ZMJU; basisOfRecord: PreservedSpecimen
- **Type status:** Other material. **Occurrence:** individualCount: 2; sex: males; lifeStage: adult; **Taxon:** scientificName: Hypotrabala castanea Holland, 1893; **Location:** continent: Africa; country: Cameroon; stateProvince: Southwest Region; locality: Bamboo Camp, Mount Cameroon; verbatimElevation: 350 m; decimalLatitude: 04.0899; decimalLongitude: 09.0517; **Identification:** identifiedBy: Vincent Maicher; dateIdentified: 2017; **Event:** samplingProtocol: Light catching; eventDate: 18/04/2015; habitat: Lowland forest disturbed by historical selective logging; **Record Level:** type: PhysicalObject; institutionID: http://grbio.org/cool/8t1f-g2z6; institutionCode: ZMJU; basisOfRecord: PreservedSpecimen

#### Distribution

**Figure 20.**
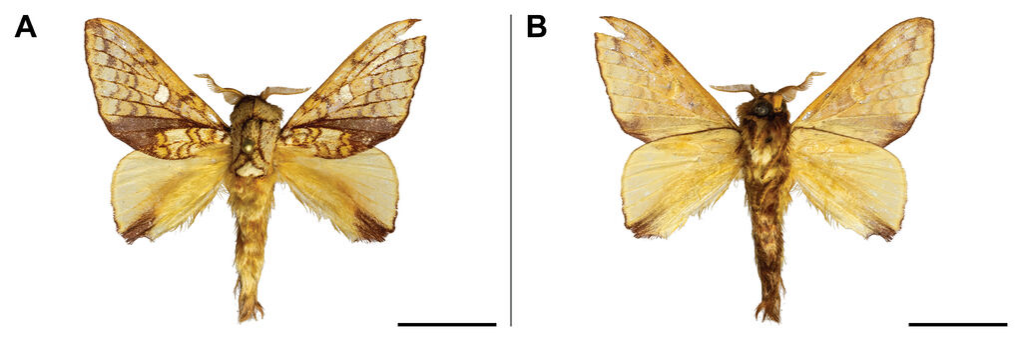
*Hypotrabala castanea* Holland, 1893. **A.** dorsal view; **B.** ventral view. The scale bar represents 1 cm.

This species was reported from Ghana, Nigeria and Gabon. It is also the first record of *Hypotrabala* genus in Cameroon. In the Mount Cameroon region, it was collected in lowland forests up to 350 m a.s.l. (Fig. 20).

### Mimopacha tripunctata

(Aurivillius, 1905)

FC4C3ACE-ED6E-5A77-8CE7-75D1262F7B64

Lasiocampidae , Lasiocampinae

#### Materials

- **Type status:** Other material. **Occurrence:** individualCount: 2; sex: males; lifeStage: adult; **Taxon:** scientificName: Mimopacha tripunctata (Aurivillius, 1905); **Location:** continent: Africa; country: Cameroon; stateProvince: Southwest Region; locality: Crater Lake, Mount Cameroon; verbatimElevation: 1,450 m; decimalLatitude: 04.1443; decimalLongitude: 09.0717; **Identification:** identifiedBy: Vincent Maicher; dateIdentified: 2017; **Event:** samplingProtocol: Light catching; eventDate: 24/11/2016; habitat: Submontane forest locally disturbed by elephants; **Record Level:** type: PhysicalObject; institutionID: http://grbio.org/cool/8t1f-g2z6; institutionCode: ZMJU; basisOfRecord: PreservedSpecimen
- **Type status:** Other material. **Occurrence:** individualCount: 2; sex: males; lifeStage: adult; **Taxon:** scientificName: Mimopacha tripunctata (Aurivillius, 1905); **Location:** continent: Africa; country: Cameroon; stateProvince: Southwest Region; locality: Elephant Camp, Mount Cameroon; verbatimElevation: 1,850 m; decimalLatitude: 04.1453; decimalLongitude: 09.0870; **Identification:** identifiedBy: Vincent Maicher; dateIdentified: 2017; **Event:** samplingProtocol: Light catching; eventDate: 18/11/2014; habitat: Montane forest locally disturbed by elephants; **Record Level:** type: PhysicalObject; institutionID: http://grbio.org/cool/8t1f-g2z6; institutionCode: ZMJU; basisOfRecord: PreservedSpecimen
- **Type status:** Other material. **Occurrence:** individualCount: 2; sex: males; lifeStage: adult; **Taxon:** scientificName: Mimopacha tripunctata (Aurivillius, 1905); **Location:** continent: Africa; country: Cameroon; stateProvince: Southwest Region; locality: Mapanja Camp, Mount Cameroon; verbatimElevation: 1,850 m; decimalLatitude: 04.1157; decimalLongitude: 09.1315; **Identification:** identifiedBy: Vincent Maicher; dateIdentified: 2017; **Event:** samplingProtocol: Light catching; eventDate: 24/10/2017; habitat: Montane forest; **Record Level:** type: PhysicalObject; institutionID: http://grbio.org/cool/8t1f-g2z6; institutionCode: ZMJU; basisOfRecord: PreservedSpecimen

#### Distribution

**Figure 21.**
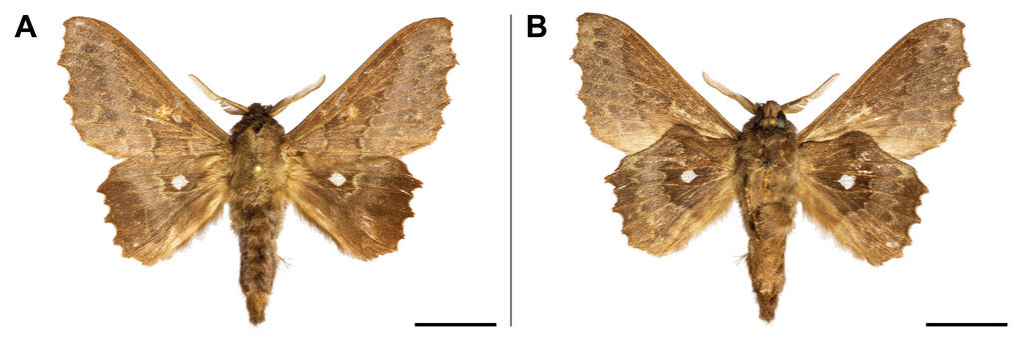
*Mimopacha tripunctata* (Aurivillius, 1905). **A.** dorsal view; **B.** ventral view. The scale bar represents 1 cm.

This species was known from the Guinean (Côte d’Ivoire and Nigeria) and Zambezian (Kenya, Tanzania and Uganda) biogeographic regions, with a large distribution gap in the Congolian biogeographic region. On Mount Cameroon, it was recorded in the submontane and montane forests between 1,450 and 1,850 m a.s.l. (Fig. 21).

### Pachytrina gliharta

Zolotuhin & Gurkovich

0FC0C261-7446-5746-9A8F-9FCFD759AE98

Lasiocampidae , Lasiocampinae

#### Materials

- **Type status:** Other material. **Occurrence:** individualCount: 1; sex: male; lifeStage: adult; **Taxon:** scientificName: Pachytrina gilharta Zolotuhin & Gurkovich, 2009; **Location:** continent: Africa; country: Cameroon; stateProvince: Southwest Region; locality: Bamboo Camp, Mount Cameroon; verbatimElevation: 350 m; decimalLatitude: 04.0899; decimalLongitude: 09.0517; **Identification:** identifiedBy: Vincent Maicher; dateIdentified: 2017; **Event:** samplingProtocol: Light catching; eventDate: 16/12/2014; habitat: Lowland forest disturbed by historical selective logging; **Record Level:** type: PhysicalObject; institutionID: http://grbio.org/cool/8t1f-g2z6; institutionCode: ZMJU; basisOfRecord: PreservedSpecimen
- **Type status:** Other material. **Occurrence:** individualCount: 1; sex: male; lifeStage: adult; **Taxon:** scientificName: Pachytrina gilharta Zolotuhin & Gurkovich, 2009; **Location:** continent: Africa; country: Cameroon; stateProvince: Southwest Region; locality: Bamboo Camp, Mount Cameroon; verbatimElevation: 350 m; decimalLatitude: 04.0899; decimalLongitude: 09.0517; **Identification:** identifiedBy: Vincent Maicher; dateIdentified: 2017; **Event:** samplingProtocol: Light catching; eventDate: 23/04/2015; habitat: Lowland forest disturbed by historical selective logging; **Record Level:** type: PhysicalObject; institutionID: http://grbio.org/cool/8t1f-g2z6; institutionCode: ZMJU; basisOfRecord: PreservedSpecimen
- **Type status:** Other material. **Occurrence:** individualCount: 1; sex: male; lifeStage: adult; **Taxon:** scientificName: Pachytrina gilharta Zolotuhin & Gurkovich, 2009; **Location:** continent: Africa; country: Cameroon; stateProvince: Southwest Region; locality: Bamboo Camp, Mount Cameroon; verbatimElevation: 350 m; decimalLatitude: 04.0899; decimalLongitude: 09.0517; **Identification:** identifiedBy: Vincent Maicher; dateIdentified: 2017; **Event:** samplingProtocol: Light catching; eventDate: 06/02/2016; habitat: Lowland forest disturbed by historical selective logging; **Record Level:** type: PhysicalObject; institutionID: http://grbio.org/cool/8t1f-g2z6; institutionCode: ZMJU; basisOfRecord: PreservedSpecimen
- **Type status:** Other material. **Occurrence:** individualCount: 1; sex: male; lifeStage: adult; **Taxon:** scientificName: Pachytrina gilharta Zolotuhin & Gurkovich, 2009; **Location:** continent: Africa; country: Cameroon; stateProvince: Southwest Region; locality: Drink Gari, Mount Cameroon; verbatimElevation: 650 m; decimalLatitude: 04.1022; decimalLongitude: 09.0630; **Identification:** identifiedBy: Vincent Maicher; dateIdentified: 2017; **Event:** samplingProtocol: Light catching; eventDate: 23/04/2015; habitat: Primary lowland forest; **Record Level:** type: PhysicalObject; institutionID: http://grbio.org/cool/8t1f-g2z6; institutionCode: ZMJU; basisOfRecord: PreservedSpecimen

#### Distribution

**Figure 22.**
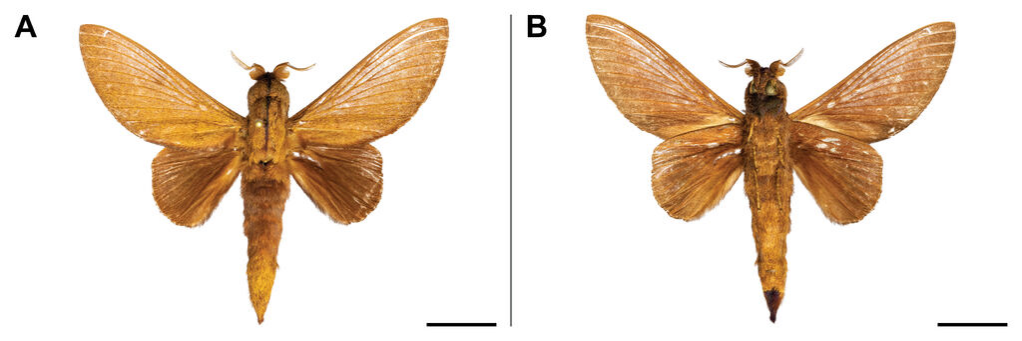
*Pachytrina gliharta* Zolotuhin & Gurkovich, 2009. **A.** dorsal view; **B.** ventral view. The scale bar represents 1 cm.

This species was already known from several countries in the Guinean and Congolian biogeographic regions, including Nigeria, Gabon and Congo bordering Cameroon. On Mount Cameroon, it was recorded in lowland forests at 350 and 650 m a.s.l. (Fig. 22).

### Archinadata aurivilliusi

(Kiriakoff, 1954)

1F34732A-5B79-51F7-B0C0-80C389BEDF9F

Notodontidae , Notodontinae

#### Materials

- **Type status:** Other material. **Occurrence:** individualCount: 1; sex: male; lifeStage: adult; **Taxon:** scientificName: Archinadata aurivilliusi (Kiriakoff, 1954); **Location:** continent: Africa; country: Cameroon; stateProvince: Southwest Region; locality: Bamboo Camp, Mount Cameroon; verbatimElevation: 350 m; decimalLatitude: 04.0899; decimalLongitude: 09.0517; **Identification:** identifiedBy: Vincent Maicher; dateIdentified: 2017; **Event:** samplingProtocol: Light catching; eventDate: 23/04/2015; habitat: Lowland forest disturbed by historical selective logging; **Record Level:** type: PhysicalObject; institutionID: http://grbio.org/cool/8t1f-g2z6; institutionCode: ZMJU; basisOfRecord: PreservedSpecimen
- **Type status:** Other material. **Occurrence:** individualCount: 5; sex: males; lifeStage: adult; **Taxon:** scientificName: Archinadata aurivilliusi (Kiriakoff, 1954); **Location:** continent: Africa; country: Cameroon; stateProvince: Southwest Region; locality: Drink Gari, Mount Cameroon; verbatimElevation: 650 m; decimalLatitude: 04.1022; decimalLongitude: 09.0630; **Identification:** identifiedBy: Vincent Maicher; dateIdentified: 2017; **Event:** samplingProtocol: Light catching; eventDate: 17/04/2015; habitat: Primary lowland forest; **Record Level:** type: PhysicalObject; institutionID: http://grbio.org/cool/8t1f-g2z6; institutionCode: ZMJU; basisOfRecord: PreservedSpecimen

#### Distribution

**Figure 23.**
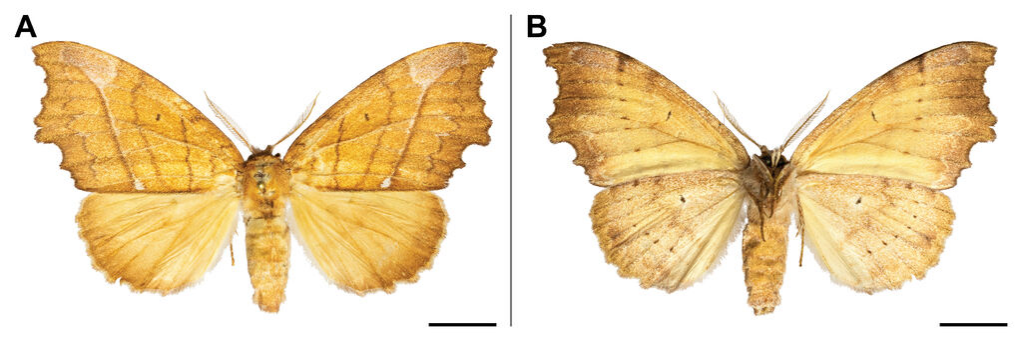
*Archinadata aurivilliusi* (Kiriakoff, 1954). **A.** dorsal view; **B.** ventral view. The scale bar represents 1 cm.

This species was known from the Guinean (Côte d’Ivoire) and Congolian (Gabon, Democratic Republic of Congo and Rwanda) biogeographic regions. It is also the first record of the *Archinadata* genus in Cameroon. On Mount Cameroon, it was recorded in the lowland forests at 350 and 650 m a.s.l. (Fig. 23).

### Brachychira punctulata

Kiriakoff, 1966

CB79E016-CD53-53B5-85C4-23C169351045

Notodontidae , Notodontinae

#### Materials

- **Type status:** Other material. **Occurrence:** individualCount: 14; sex: males; lifeStage: adult; **Taxon:** scientificName: Brachychira punctulata Kiriakoff, 1966; **Location:** continent: Africa; country: Cameroon; stateProvince: Southwest Region; locality: Bamboo Camp, Mount Cameroon; verbatimElevation: 350 m; decimalLatitude: 04.0899; decimalLongitude: 09.0517; **Identification:** identifiedBy: Vincent Maicher; dateIdentified: 2017; **Event:** samplingProtocol: Light catching; eventDate: 16/12/2014; habitat: Lowland forest disturbed by historical selective logging; **Record Level:** type: PhysicalObject; institutionID: http://grbio.org/cool/8t1f-g2z6; institutionCode: ZMJU; basisOfRecord: PreservedSpecimen
- **Type status:** Other material. **Occurrence:** individualCount: 1; sex: male; lifeStage: adult; **Taxon:** scientificName: Brachychira punctulata Kiriakoff, 1966; **Location:** continent: Africa; country: Cameroon; stateProvince: Southwest Region; locality: Bamboo Camp, Mount Cameroon; verbatimElevation: 350 m; decimalLatitude: 04.0899; decimalLongitude: 09.0517; **Identification:** identifiedBy: Vincent Maicher; dateIdentified: 2017; **Event:** samplingProtocol: Light catching; eventDate: 22/04/2015; habitat: Lowland forest disturbed by historical selective logging; **Record Level:** type: PhysicalObject; institutionID: http://grbio.org/cool/8t1f-g2z6; institutionCode: ZMJU; basisOfRecord: PreservedSpecimen
- **Type status:** Other material. **Occurrence:** individualCount: 7; sex: 6 males, 1 female; lifeStage: adult; **Taxon:** scientificName: Brachychira punctulata Kiriakoff, 1966; **Location:** continent: Africa; country: Cameroon; stateProvince: Southwest Region; locality: Bamboo Camp, Mount Cameroon; verbatimElevation: 350 m; decimalLatitude: 04.0899; decimalLongitude: 09.0517; **Identification:** identifiedBy: Vincent Maicher; dateIdentified: 2017; **Event:** samplingProtocol: Light catching; eventDate: 08/02/2016; habitat: Lowland forest disturbed by historical selective logging; **Record Level:** type: PhysicalObject; institutionID: http://grbio.org/cool/8t1f-g2z6; institutionCode: ZMJU; basisOfRecord: PreservedSpecimen
- **Type status:** Other material. **Occurrence:** individualCount: 20; sex: males; lifeStage: adult; **Taxon:** scientificName: Brachychira punctulata Kiriakoff, 1966; **Location:** continent: Africa; country: Cameroon; stateProvince: Southwest Region; locality: Drink Gari, Mount Cameroon; verbatimElevation: 650 m; decimalLatitude: 04.1022; decimalLongitude: 09.0630; **Identification:** identifiedBy: Vincent Maicher; dateIdentified: 2017; **Event:** samplingProtocol: Light catching; eventDate: 05/02/2016; habitat: Primary lowland forest; **Record Level:** type: PhysicalObject; institutionID: http://grbio.org/cool/8t1f-g2z6; institutionCode: ZMJU; basisOfRecord: PreservedSpecimen
- **Type status:** Other material. **Occurrence:** individualCount: 5; sex: males; lifeStage: adult; **Taxon:** scientificName: Brachychira punctulata Kiriakoff, 1966; **Location:** continent: Africa; country: Cameroon; stateProvince: Southwest Region; locality: Drink Gari, Mount Cameroon; verbatimElevation: 650 m; decimalLatitude: 04.1022; decimalLongitude: 09.0630; **Identification:** identifiedBy: Vincent Maicher; dateIdentified: 2017; **Event:** samplingProtocol: Light catching; eventDate: 09/12/2014; habitat: Primary lowland forest; **Record Level:** type: PhysicalObject; institutionID: http://grbio.org/cool/8t1f-g2z6; institutionCode: ZMJU; basisOfRecord: PreservedSpecimen
- **Type status:** Other material. **Occurrence:** individualCount: 7; sex: males; lifeStage: adult; **Taxon:** scientificName: Brachychira punctulata Kiriakoff, 1966; **Location:** continent: Africa; country: Cameroon; stateProvince: Southwest Region; locality: Drink Gari, Mount Cameroon; verbatimElevation: 650 m; decimalLatitude: 04.1022; decimalLongitude: 09.0630; **Identification:** identifiedBy: Vincent Maicher; dateIdentified: 2017; **Event:** samplingProtocol: Light catching; eventDate: 20/04/2015; habitat: Primary lowland forest; **Record Level:** type: PhysicalObject; institutionID: http://grbio.org/cool/8t1f-g2z6; institutionCode: ZMJU; basisOfRecord: PreservedSpecimen
- **Type status:** Other material. **Occurrence:** individualCount: 5; sex: males; lifeStage: adult; **Taxon:** scientificName: Brachychira punctulata Kiriakoff, 1966; **Location:** continent: Africa; country: Cameroon; stateProvince: Southwest Region; locality: PlanteCam Camp, Mount Cameroon; verbatimElevation: 1,100 m; decimalLatitude: 04.1175; decimalLongitude: 09.0709; **Identification:** identifiedBy: Vincent Maicher; dateIdentified: 2017; **Event:** samplingProtocol: Light catching; eventDate: 11/04/2015; habitat: Upland forest locally disturbed by elephants; **Record Level:** type: PhysicalObject; institutionID: http://grbio.org/cool/8t1f-g2z6; institutionCode: ZMJU; basisOfRecord: PreservedSpecimen
- **Type status:** Other material. **Occurrence:** individualCount: 4; sex: males; lifeStage: adult; **Taxon:** scientificName: Brachychira punctulata Kiriakoff, 1966; **Location:** continent: Africa; country: Cameroon; stateProvince: Southwest Region; locality: PlanteCam Camp, Mount Cameroon; verbatimElevation: 1,100 m; decimalLatitude: 04.1175; decimalLongitude: 09.0709; **Identification:** identifiedBy: Vincent Maicher; dateIdentified: 2017; **Event:** samplingProtocol: Light catching; eventDate: 01/02/2016; habitat: Upland forest locally disturbed by elephants; **Record Level:** type: PhysicalObject; institutionID: http://grbio.org/cool/8t1f-g2z6; institutionCode: ZMJU; basisOfRecord: PreservedSpecimen

#### Distribution

**Figure 24.**
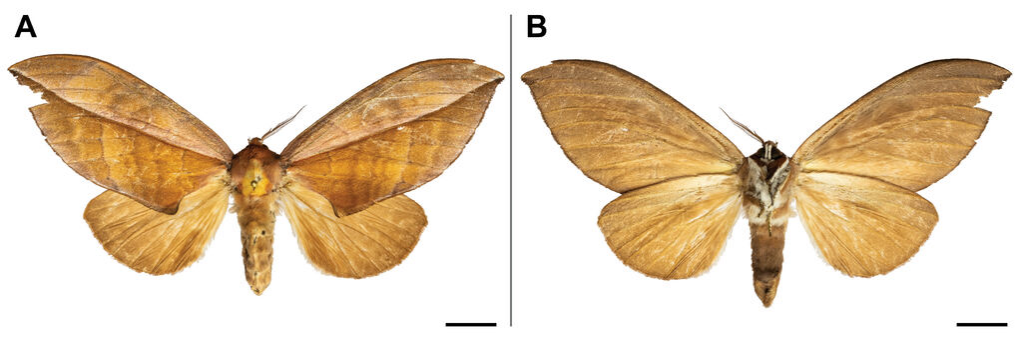
*Brachychira punctulata* Kiriakoff, 1966. **A.** dorsal view; **B.** ventral view. The scale bar represents 1 cm.

This species was considered endemic to Gabon. Our record in Cameroon extended its distribution range into the Guinean biogeographic region. On Mount Cameroon, it occurred in lowland and upland forests between 350 and 1,100 m a.s.l. (Fig. 24).

### Gargettoscrancia albolineata

(Strand, 1912)

43016649-A5C8-5ED7-917D-8BCC1178889C

Notodontidae , Notodontinae

#### Materials

- **Type status:** Other material. **Occurrence:** individualCount: 1; sex: male; lifeStage: adult; **Taxon:** scientificName: Gargettoscrancia albolineata (Strand, 1912); **Location:** continent: Africa; country: Cameroon; stateProvince: Southwest Region; locality: Bamboo Camp, Mount Cameroon; verbatimElevation: 350 m; decimalLatitude: 04.0899; decimalLongitude: 09.0517; **Identification:** identifiedBy: Vincent Maicher; dateIdentified: 2017; **Event:** samplingProtocol: Light catching; eventDate: 17/04/2015; habitat: Lowland forest disturbed by historical selective logging; **Record Level:** type: PhysicalObject; institutionID: http://grbio.org/cool/8t1f-g2z6; institutionCode: ZMJU; basisOfRecord: PreservedSpecimen
- **Type status:** Other material. **Occurrence:** individualCount: 1; sex: female; lifeStage: adult; **Taxon:** scientificName: Gargettoscrancia albolineata (Strand, 1912); **Location:** continent: Africa; country: Cameroon; stateProvince: Southwest Region; locality: Drink Gari, Mount Cameroon; verbatimElevation: 650 m; decimalLatitude: 04.1022; decimalLongitude: 09.0630; **Identification:** identifiedBy: Vincent Maicher; dateIdentified: 2017; **Event:** samplingProtocol: Light catching; eventDate: 22/04/2015; habitat: Primary lowland forest; **Record Level:** type: PhysicalObject; institutionID: http://grbio.org/cool/8t1f-g2z6; institutionCode: ZMJU; basisOfRecord: PreservedSpecimen
- **Type status:** Other material. **Occurrence:** individualCount: 2; sex: males; lifeStage: adult; **Taxon:** scientificName: Gargettoscrancia albolineata (Strand, 1912); **Location:** continent: Africa; country: Cameroon; stateProvince: Southwest Region; locality: PlanteCam Camp, Mount Cameroon; verbatimElevation: 1,100 m; decimalLatitude: 04.1175; decimalLongitude: 09.0709; **Identification:** identifiedBy: Vincent Maicher; dateIdentified: 2017; **Event:** samplingProtocol: Light catching; eventDate: 13/04/2015; habitat: Upland forest locally disturbed by elephants; **Record Level:** type: PhysicalObject; institutionID: http://grbio.org/cool/8t1f-g2z6; institutionCode: ZMJU; basisOfRecord: PreservedSpecimen
- **Type status:** Other material. **Occurrence:** individualCount: 1; sex: male; lifeStage: adult; **Taxon:** scientificName: Gargettoscrancia albolineata (Strand, 1912); **Location:** continent: Africa; country: Cameroon; stateProvince: Southwest Region; locality: Crater Lake, Mount Cameroon; verbatimElevation: 1,450 m; decimalLatitude: 04.1443; decimalLongitude: 09.0717; **Identification:** identifiedBy: Vincent Maicher; dateIdentified: 2017; **Event:** samplingProtocol: Light catching; eventDate: 17/02/2017; habitat: Submontane forest locally disturbed by elephants; **Record Level:** type: PhysicalObject; institutionID: http://grbio.org/cool/8t1f-g2z6; institutionCode: ZMJU; basisOfRecord: PreservedSpecimen

#### Distribution

**Figure 25.**
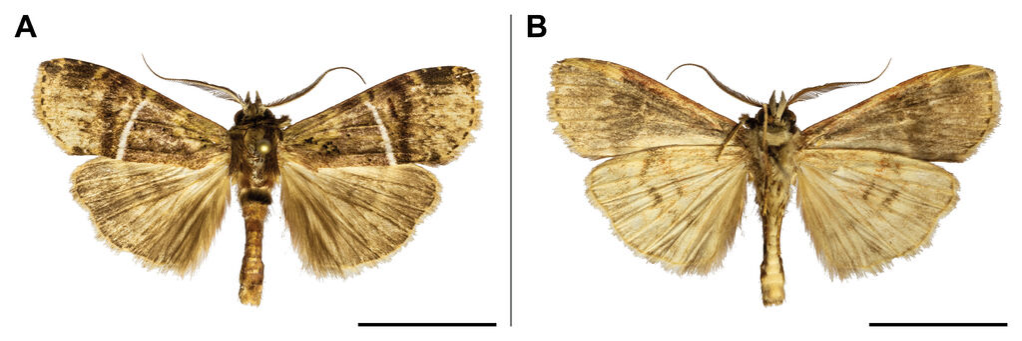
*Gargettoscrancia albolineata* (Strand, 1912). **A.** dorsal view; **B.** ventral view. The scale bar represents 1 cm.

This species was known from the Guinean (Ivory Coast) and Congolian (Equatorial Guinea and the Democratic Republic of the Congo) biogeographic regions. It is also the first record of the *Gargettoscrania* genus in Cameroon. On Mount Cameroon, it was collected in lowland (350 m a.s.l.) to submontane (1,450 m a.s.l.) forests (Fig. 25).

### Pseudobarobata denticulata

Kiriakoff, 1966

C67707D6-19D8-5B39-9E89-6117F5EC00CC

Notodontidae , Notodontinae

#### Materials

- **Type status:** Other material. **Occurrence:** individualCount: 1; sex: female; lifeStage: adult; **Taxon:** scientificName: Pseudobarobata denticulata Kiriakoff, 1966; **Location:** continent: Africa; country: Cameroon; stateProvince: Southwest Region; locality: Dikolo Peninsula, Bimbia-Bonadikombo Community Forest; verbatimElevation: 30 m; decimalLatitude: 03.9818; decimalLongitude: 09.2625; **Identification:** identifiedBy: Vincent Maicher; dateIdentified: 2017; **Event:** samplingProtocol: Light catching; eventDate: 14/01/2016; habitat: Coastal forest; **Record Level:** type: PhysicalObject; institutionID: http://grbio.org/cool/8t1f-g2z6; institutionCode: ZMJU; basisOfRecord: PreservedSpecimen
- **Type status:** Other material. **Occurrence:** individualCount: 1; sex: male; lifeStage: adult; **Taxon:** scientificName: Pseudobarobata denticulata Kiriakoff, 1966; **Location:** continent: Africa; country: Cameroon; stateProvince: Southwest Region; locality: Bamboo Camp, Mount Cameroon; verbatimElevation: 350 m; decimalLatitude: 04.0899; decimalLongitude: 09.0517; **Identification:** identifiedBy: Vincent Maicher; dateIdentified: 2017; **Event:** samplingProtocol: Light catching; eventDate: 13/12/2014; habitat: Lowland forest disturbed by historical selective logging; **Record Level:** type: PhysicalObject; institutionID: http://grbio.org/cool/8t1f-g2z6; institutionCode: ZMJU; basisOfRecord: PreservedSpecimen
- **Type status:** Other material. **Occurrence:** individualCount: 4; sex: males; lifeStage: adult; **Taxon:** scientificName: Pseudobarobata denticulata Kiriakoff, 1966; **Location:** continent: Africa; country: Cameroon; stateProvince: Southwest Region; locality: Bamboo Camp, Mount Cameroon; verbatimElevation: 350 m; decimalLatitude: 04.0899; decimalLongitude: 09.0517; **Identification:** identifiedBy: Vincent Maicher; dateIdentified: 2017; **Event:** samplingProtocol: Light catching; eventDate: 21/04/2015; habitat: Lowland forest disturbed by historical selective logging; **Record Level:** type: PhysicalObject; institutionID: http://grbio.org/cool/8t1f-g2z6; institutionCode: ZMJU; basisOfRecord: PreservedSpecimen
- **Type status:** Other material. **Occurrence:** individualCount: 1; sex: male; lifeStage: adult; **Taxon:** scientificName: Pseudobarobata denticulata Kiriakoff, 1966; **Location:** continent: Africa; country: Cameroon; stateProvince: Southwest Region; locality: Bamboo Camp, Mount Cameroon; verbatimElevation: 350 m; decimalLatitude: 04.0899; decimalLongitude: 09.0517; **Identification:** identifiedBy: Vincent Maicher; dateIdentified: 2017; **Event:** samplingProtocol: Light catching; eventDate: 11/02/2016; habitat: Lowland forest disturbed by historical selective logging; **Record Level:** type: PhysicalObject; institutionID: http://grbio.org/cool/8t1f-g2z6; institutionCode: ZMJU; basisOfRecord: PreservedSpecimen
- **Type status:** Other material. **Occurrence:** individualCount: 1; sex: male; lifeStage: adult; **Taxon:** scientificName: Pseudobarobata denticulata Kiriakoff, 1966; **Location:** continent: Africa; country: Cameroon; stateProvince: Southwest Region; locality: Crater Lake, Mount Cameroon; verbatimElevation: 1,450 m; decimalLatitude: 04.1443; decimalLongitude: 09.0717; **Identification:** identifiedBy: Vincent Maicher; dateIdentified: 2017; **Event:** samplingProtocol: Light catching; eventDate: 20/11/2016; habitat: Submontane forest locally disturbed by elephants; **Record Level:** type: PhysicalObject; institutionID: http://grbio.org/cool/8t1f-g2z6; institutionCode: ZMJU; basisOfRecord: PreservedSpecimen

#### Distribution

**Figure 26.**
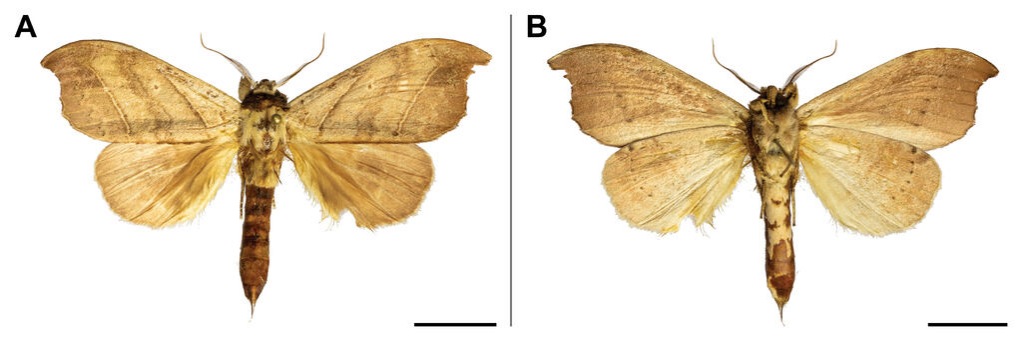
*Pseudobarobata denticulata* Kiriakoff, 1966. **A.** dorsal view; **B.** ventral view. The scale bar represents 1 cm.

This species was known from Gabon, Central African Republic and Tanzania. Our record from Cameroon thus broadened its known distribution westwards, as well as into the Guinean biogeographic region. In the Mount Cameroon region, it was collected in lowland forests (30 and 350 m a.s.l.), although one specimen was recorded in submontane forest (1,450 m a.s.l.) (Fig. 26).

### Pseudobarobata integra

Kiriakoff, 1966

7E7B045F-7DDF-5A98-B635-E1F78619E839

Notodontidae , Notodontinae

#### Materials

- **Type status:** Other material. **Occurrence:** individualCount: 2; sex: males; lifeStage: adult; **Taxon:** scientificName: Pseudobarobata integra Kiriakoff, 1966; **Location:** continent: Africa; country: Cameroon; stateProvince: Southwest Region; locality: Bamboo Camp, Mount Cameroon; verbatimElevation: 350 m; decimalLatitude: 04.0899; decimalLongitude: 09.0517; **Identification:** identifiedBy: Vincent Maicher; dateIdentified: 2017; **Event:** samplingProtocol: Light catching; eventDate: 12/12/2014; habitat: Lowland forest disturbed by historical selective logging; **Record Level:** type: PhysicalObject; institutionID: http://grbio.org/cool/8t1f-g2z6; institutionCode: ZMJU; basisOfRecord: PreservedSpecimen
- **Type status:** Other material. **Occurrence:** individualCount: 1; sex: male; lifeStage: adult; **Taxon:** scientificName: Pseudobarobata integra Kiriakoff, 1966; **Location:** continent: Africa; country: Cameroon; stateProvince: Southwest Region; locality: Drink Gari, Mount Cameroon; verbatimElevation: 650 m; decimalLatitude: 04.1022; decimalLongitude: 09.0630; **Identification:** identifiedBy: Vincent Maicher; dateIdentified: 2017; **Event:** samplingProtocol: Light catching; eventDate: 27/11/2014; habitat: Primary lowland forest; **Record Level:** type: PhysicalObject; institutionID: http://grbio.org/cool/8t1f-g2z6; institutionCode: ZMJU; basisOfRecord: PreservedSpecimen

#### Distribution

**Figure 27.**
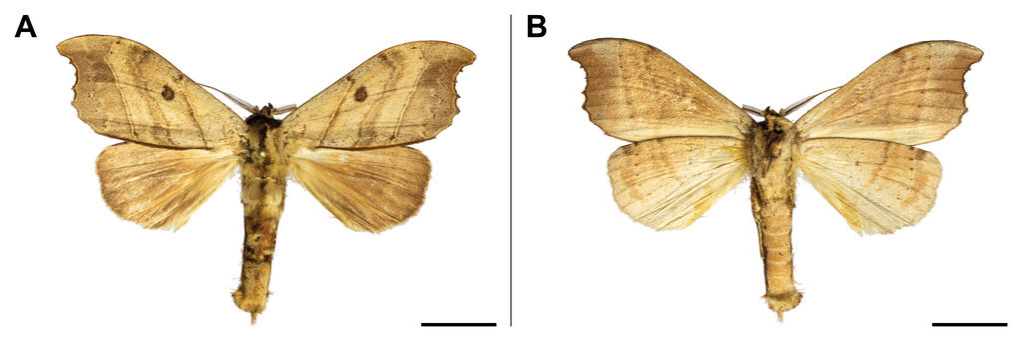
*Pseudobarobata integra* Kiriakoff, 1966. **A.** dorsal view; **B.** ventral view. The scale bar represents 1 cm.

This species was reported from the Congolian biogeographic region only (Central African Republic and Gabon). Our record extended its known distribution to the Guinean biogeographic region. On Mount Cameroon, it was recorded in lowland forests at 350 and 650 m a.s.l. (Fig. 27).

### Borbo borbonica

(Boisduval, 1833)

4B4DA133-59BB-5665-9A97-A6C1340217B3

Hesperiidae , Hesperiinae

#### Materials

- **Type status:** Other material. **Occurrence:** individualCount: 1; lifeStage: adult; **Taxon:** scientificName: Borbo borbonica (Boisduval, 1833); **Location:** continent: Africa; country: Cameroon; stateProvince: Southwest Region; locality: Dikolo Peninsula, Bimbia-Bonadikombo Community Forest; verbatimElevation: 30 m; decimalLatitude: 03.9818; decimalLongitude: 09.2625; **Identification:** identifiedBy: Szabolcs Sáfián; dateIdentified: 2015; **Event:** samplingProtocol: Butterfly net; eventDate: 30/12/2014; habitat: Coastal forest; **Record Level:** type: PhysicalObject; institutionID: http://grbio.org/cool/8t1f-g2z6; institutionCode: ZMJU; basisOfRecord: PreservedSpecimen
- **Type status:** Other material. **Occurrence:** individualCount: 1; lifeStage: adult; **Taxon:** scientificName: Borbo borbonica (Boisduval, 1833); **Location:** continent: Africa; country: Cameroon; stateProvince: Southwest Region; locality: Crater Lake, Mount Cameroon; verbatimElevation: 1,450 m; decimalLatitude: 04.1443; decimalLongitude: 09.0717; **Identification:** identifiedBy: Szabolcs Sáfián; dateIdentified: 2015; **Event:** samplingProtocol: Butterfly net; eventDate: 26/04/2017; habitat: Submontane forest locally disturbed by elephants; **Record Level:** type: PhysicalObject; institutionID: http://grbio.org/cool/8t1f-g2z6; institutionCode: ZMJU; basisOfRecord: PreservedSpecimen

#### Distribution

**Figure 28.**
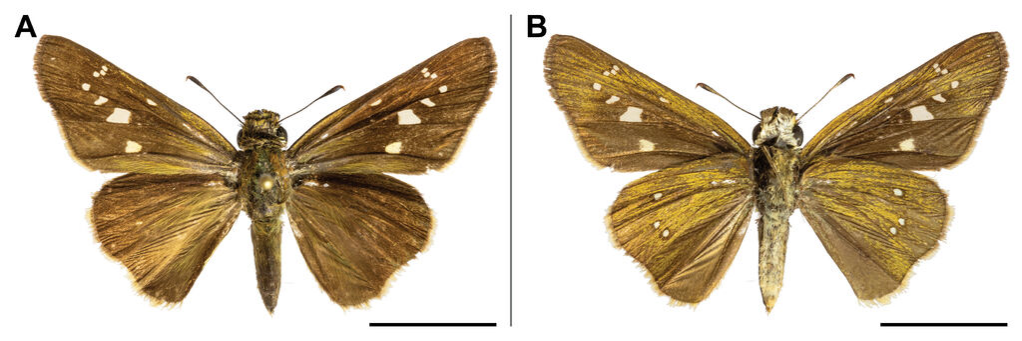
*Borbo borbonica* (Boisduval, 1833). **A.** dorsal view; **B.** ventral view. The scale bar represents 1 cm.

The nominotypical subspecies is relatively common in the Guinean biogeographic zone (most countries along the seashore between Mauritania and Nigeria) and from the Southern African region and Madagascar. Our Cameroonian record thus extended its distribution to the easternmost edge of the Guinean biogeographic zone. In the Mount Cameroon region, it was recorded from coastal (30 m a.s.l.) and submontane forests (1,450 m a.s.l.) (Fig. 28).

### Meza mabillei

(Holland, 1893)

2331368A-6959-5F2B-93E9-6E2A96CE99A7

Hesperiidae , Hesperiinae

#### Materials

- **Type status:** Other material. **Occurrence:** individualCount: 1; lifeStage: adult; **Taxon:** scientificName: Meza mabillei (Holland, 1893); **Location:** continent: Africa; country: Cameroon; stateProvince: Southwest Region; locality: Dikolo Peninsula, Bimbia-Bonadikombo Community Forest; verbatimElevation: 30 m; decimalLatitude: 03.9818; decimalLongitude: 09.2625; **Identification:** identifiedBy: Szabolcs Sáfián | Robert Tropek; dateIdentified: 2015; **Event:** samplingProtocol: Butterfly net; eventDate: 09/05/2015; habitat: Coastal forest; **Record Level:** type: PhysicalObject; institutionID: http://grbio.org/cool/8t1f-g2z6; institutionCode: ZMJU; basisOfRecord: PreservedSpecimen
- **Type status:** Other material. **Occurrence:** individualCount: 1; lifeStage: adult; **Taxon:** scientificName: Meza mabillei (Holland, 1893); **Location:** continent: Africa; country: Cameroon; stateProvince: Southwest Region; locality: Radio Hill, Bimbia village; verbatimElevation: 220 m; decimalLatitude: 03.9666; decimalLongitude: 09.2411; **Identification:** identifiedBy: Szabolcs Sáfián | Robert Tropek; dateIdentified: 2015; **Event:** samplingProtocol: Butterfly net; eventDate: 30/12/2014; habitat: Hilltop with disturbed lowland forest; **Record Level:** type: PhysicalObject; institutionID: http://grbio.org/cool/8t1f-g2z6; institutionCode: ZMJU; basisOfRecord: PreservedSpecimen

#### Distribution

**Figure 29.**
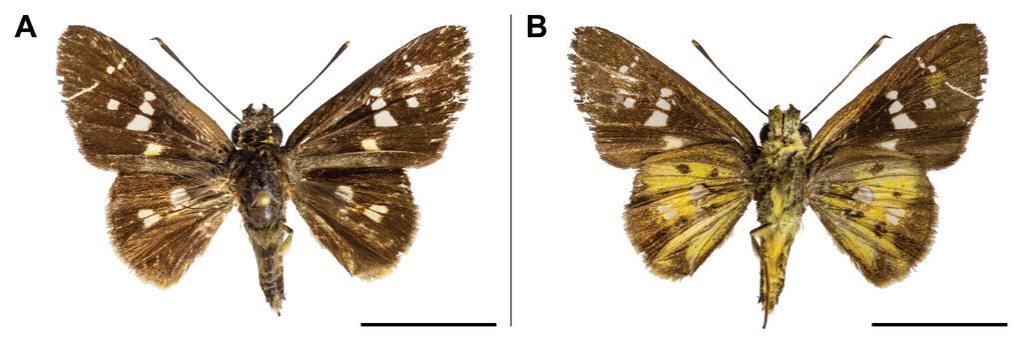
*Meza mabillei* (Holland, 1893). **A.** dorsal view; **B.** ventral view. The scale bar represents 1 cm.

This species was known to be widely distributed in the Guinean biogeographic region (most countries from Guinea to Nigeria) and in Gabon. In the Mount Cameroon region, it was collected in the two lowest localities (30 and 220 m a.s.l.) (Fig. 29).

### Acraea macaria

(Faricius, 1793)

4C729E00-DAA1-54E3-A71C-A9BFF9C0D1A4

Nymphalidae , Heliconiinae

#### Materials

- **Type status:** Other material. **Occurrence:** individualCount: 1; lifeStage: adult; **Taxon:** scientificName: Acraea macaria (Faricius, 1793); **Location:** continent: Africa; country: Cameroon; stateProvince: Southwest Region; locality: Drink Gari, Mount Cameroon; verbatimElevation: 650 m; decimalLatitude: 04.1022; decimalLongitude: 09.0630; **Identification:** identifiedBy: Szabolcs Sáfián | Robert Tropek; dateIdentified: 2015; **Event:** samplingProtocol: Butterfly net; eventDate: 01/12/2014; habitat: Primary lowland forest; **Record Level:** type: PhysicalObject; institutionID: http://grbio.org/cool/8t1f-g2z6; institutionCode: ZMJU; basisOfRecord: PreservedSpecimen
- **Type status:** Other material. **Occurrence:** individualCount: 1; lifeStage: adult; **Taxon:** scientificName: Acraea macaria (Faricius, 1793); **Location:** continent: Africa; country: Cameroon; stateProvince: Southwest Region; locality: Bamboo Camp, Mount Cameroon; verbatimElevation: 350 m; decimalLatitude: 04.0899; decimalLongitude: 09.0517; **Identification:** identifiedBy: Szabolcs Sáfián | Robert Tropek; dateIdentified: 2015; **Event:** samplingProtocol: Butterfly net; eventDate: 26/04/2015; habitat: Lowland forest disturbed by historical selective logging; **Record Level:** type: PhysicalObject; institutionID: http://grbio.org/cool/8t1f-g2z6; institutionCode: ZMJU; basisOfRecord: PreservedSpecimen

#### Distribution

*A. macaria* was known from the western part of the Guinean biogeographic region only (most countries along the seashore between Senegal and Ghana). Our record from Cameroon therefore extended its distribution for over 1,000 km westwards to the easternmost edge of the Guinean biogeographic region. In the Mount Cameroon region, it was collected in the two lowest localities (30 and 220 m a.s.l.).

### Telchinia encedana

(Pierre, 1976)

7AA00D36-CF19-5FAC-B145-6F58F5454586

Nymphalidae , Heliconiinae

#### Materials

- **Type status:** Other material. **Occurrence:** individualCount: 1; sex: male; lifeStage: adult; **Taxon:** scientificName: Telchinia encedana (Pierre, 1976); **Location:** continent: Africa; country: Cameroon; stateProvince: Northwest Region; locality: Mendong Buo, Big Babanki, Bamenda Highlands; verbatimElevation: 2,200 m; decimalLatitude: 06.0921; decimalLongitude: 10.2987; **Identification:** identifiedBy: Robert Tropek; dateIdentified: 2017; **Event:** samplingProtocol: Butterfly net; eventDate: 30/11/2016; habitat: Mosaic of mountain forest remnants, forest clearings dominated by Pteridium aquilinum, submontane grasslands maintained by cattle grazing and species‐rich scrub vegetation along streams; **Record Level:** type: PhysicalObject; institutionID: http://grbio.org/cool/8t1f-g2z6; institutionCode: ZMJU; basisOfRecord: PreservedSpecimen

#### Distribution

**Figure 30.**
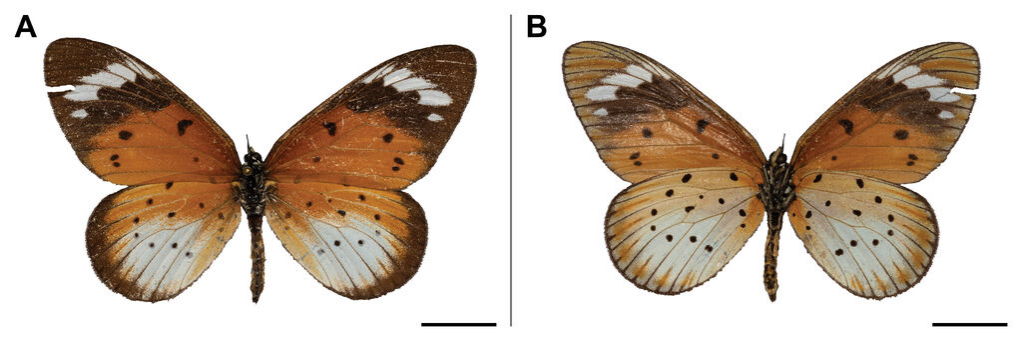
*Telchinia encedana* (Pierre, 1976). **A.** dorsal view; **B.** ventral view. The scale bar represents 1 cm.

This relatively widespread species was recorded from numerous countries of the Guinean (from Senegal to Cameroon) and Congolian (Democratic Republic of the Congo) biogeographic regions, but also from the Ethiopian, Somalian, Zambezian and Shaba regions. In Cameroon, its only record was published by Aurivillius (1905) more than a century ago, from the Adamawa Province. Our recent record therefore confirmed its presence in the country and extended the species’ distribution range to the Northwest Province (Fig. 30).

### Euphaedra temeraria

Hecq, 2007

ED4E9A59-F577-500F-B726-8DCDF533B52C

Nymphalidae , Limenitidinae

#### Materials

- **Type status:** Other material. **Occurrence:** individualCount: 72; lifeStage: adult; **Taxon:** scientificName: Euphaedra temeraria Hecq, 2007; **Location:** continent: Africa; country: Cameroon; stateProvince: Southwest Region; locality: Bamboo Camp, Mount Cameroon; verbatimElevation: 350 m; decimalLatitude: 04.0899; decimalLongitude: 09.0517; **Identification:** identifiedBy: Szabolcs Sáfián; dateIdentified: 2015; **Event:** samplingProtocol: Bait trap; eventDate: 07/12/2014; habitat: Lowland forest disturbed by historical selective logging; **Record Level:** type: PhysicalObject; institutionID: http://grbio.org/cool/8t1f-g2z6; institutionCode: ZMJU; basisOfRecord: PreservedSpecimen
- **Type status:** Other material. **Occurrence:** individualCount: 53; lifeStage: adult; **Taxon:** scientificName: Euphaedra temeraria Hecq, 2007; **Location:** continent: Africa; country: Cameroon; stateProvince: Southwest Region; locality: Drink Gari, Mount Cameroon; verbatimElevation: 650 m; decimalLatitude: 04.1022; decimalLongitude: 09.0630; **Identification:** identifiedBy: Szabolcs Sáfián; dateIdentified: 2015; **Event:** samplingProtocol: Bait trap; eventDate: 02/12/2014; habitat: Primary lowland forest; **Record Level:** type: PhysicalObject; institutionID: http://grbio.org/cool/8t1f-g2z6; institutionCode: ZMJU; basisOfRecord: PreservedSpecimen
- **Type status:** Other material. **Occurrence:** individualCount: 1; lifeStage: adult; **Taxon:** scientificName: Euphaedra temeraria Hecq, 2007; **Location:** continent: Africa; country: Cameroon; stateProvince: Southwest Region; locality: PlanteCam Camp, Mount Cameroon; verbatimElevation: 1,100 m; decimalLatitude: 04.1175; decimalLongitude: 09.0709; **Identification:** identifiedBy: Szabolcs Sáfián; dateIdentified: 2015; **Event:** samplingProtocol: Bait trap; eventDate: 14/12/2014; habitat: Upland forest locally disturbed by elephants; **Record Level:** type: PhysicalObject; institutionID: http://grbio.org/cool/8t1f-g2z6; institutionCode: ZMJU; basisOfRecord: PreservedSpecimen

#### Distribution

**Figure 31.**
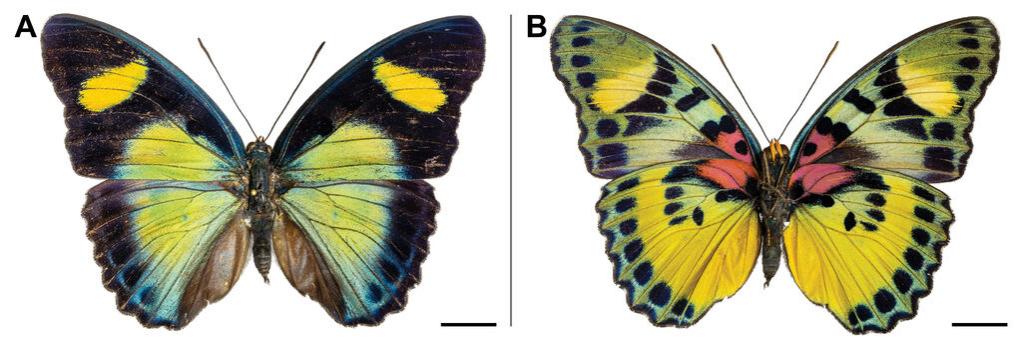
*Euphaedra temeraria* Hecq, 2007. **A.** dorsal view; **B.** ventral view. The scale bar represents 1 cm.

This species was known from Equatorial Guinea and Gabon, both in the Congolian biogeographic region. Our record thus extended its distribution range northwards and evidenced its occurrence in the Guinean biogeographic region. On Mount Cameroon, it was collected mostly in the lowland forests at 350 and 650 m a.s.l.; one specimen was caught also in the upland forest at 1,100 m a.s.l. (Fig. 31).

### Neptis metella

(Doubleday, [1850])

C733CF22-A25C-5DC4-99D5-CBAB0EC90C56

Nymphalidae , Limenitidinae

#### Materials

- **Type status:** Other material. **Occurrence:** individualCount: 1; lifeStage: adult; **Taxon:** scientificName: Neptis metella (Doubleday, [1850]); **Location:** continent: Africa; country: Cameroon; stateProvince: Southwest Region; locality: Radio Hill, Bimbia village; verbatimElevation: 220 m; decimalLatitude: 03.9666; decimalLongitude: 09.2411; **Identification:** identifiedBy: Szabolcs Sáfián | Robert Tropek; dateIdentified: 2015; **Event:** samplingProtocol: Butterfly net; eventDate: 31/12/2014; habitat: Hilltop with disturbed lowland forest; **Record Level:** type: PhysicalObject; institutionID: http://grbio.org/cool/8t1f-g2z6; institutionCode: ZMJU; basisOfRecord: PreservedSpecimen
- **Type status:** Other material. **Occurrence:** individualCount: 1; lifeStage: adult; **Taxon:** scientificName: Neptis metella (Doubleday, [1850]); **Location:** continent: Africa; country: Cameroon; stateProvince: Southwest Region; locality: Radio Hill, Bimbia village; verbatimElevation: 220 m; decimalLatitude: 03.9666; decimalLongitude: 09.2411; **Identification:** identifiedBy: Szabolcs Sáfián | Robert Tropek; dateIdentified: 2015; **Event:** samplingProtocol: Butterfly net; eventDate: 08/01/2015; habitat: Hilltop with disturbed lowland forest; **Record Level:** type: PhysicalObject; institutionID: http://grbio.org/cool/8t1f-g2z6; institutionCode: ZMJU; basisOfRecord: PreservedSpecimen
- **Type status:** Other material. **Occurrence:** individualCount: 1; lifeStage: adult; **Taxon:** scientificName: Neptis metella (Doubleday, [1850]); **Location:** continent: Africa; country: Cameroon; stateProvince: Northwest Region; locality: Lake Oku, Mount Oku; verbatimElevation: 2,200 m; decimalLatitude: 06.1924; decimalLongitude: 10.4616; **Identification:** identifiedBy: Robert Tropek; dateIdentified: 2015; **Event:** samplingProtocol: Butterfly net; eventDate: 24/12/2009; habitat: Montane forest; **Record Level:** type: PhysicalObject; institutionID: http://grbio.org/cool/06zb-203s; institutionCode: IECA; basisOfRecord: PreservedSpecimen
- **Type status:** Other material. **Occurrence:** individualCount: 1; lifeStage: adult; **Taxon:** scientificName: Neptis metella (Doubleday, [1850]); **Location:** continent: Africa; country: Cameroon; stateProvince: Centre Region; locality: Ebogo Forest; verbatimElevation: 660 m; decimalLatitude: 03.3880; decimalLongitude: 11.4700; **Identification:** identifiedBy: Robert Tropek; dateIdentified: 2015; **Event:** samplingProtocol: Butterfly net; eventDate: 04/01/2012; habitat: Disturbed lowland forest; **Record Level:** type: PhysicalObject; institutionID: http://grbio.org/cool/06zb-203s; institutionCode: IECA; basisOfRecord: PreservedSpecimen

#### Distribution

**Figure 32.**
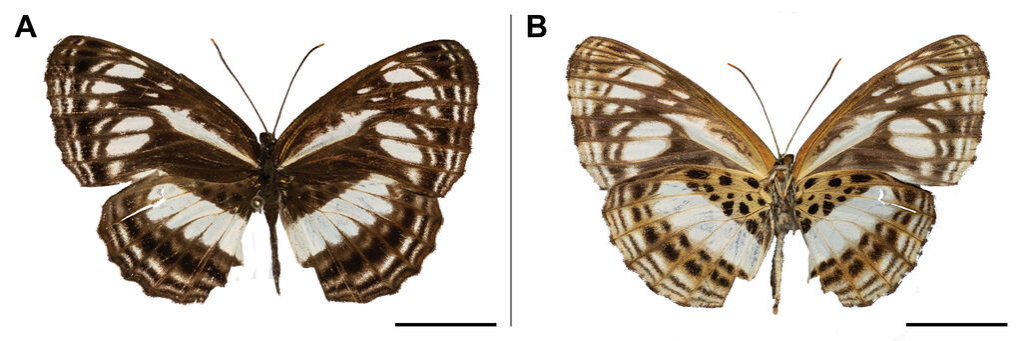
*Neptis metella* (Doubleday, [1850]). **A.** dorsal view; **B.** ventral view. The scale bar represents 1 cm.

This species was previously known from the Guinean (from Guinea to Nigeria), Shaba, Zambezian and Sudanian biogeographic regions. Our record in Cameroon partly filled the gap in its known distribution. It was known as a relatively common species in many disturbed lowland forests in the surrounding countries (Larsen 2005), thus its occurrence in Cameroon is not surprising. Three of the four reported specimens were collected in disturbed lowland forests (220 and 660 m a.s.l.), corresponding to the known species’ habitats (Larsen 2005). On the other hand, its occurrence at 2,200 m a.s.l. has evidenced the species as a habitat generalist (Fig. 32).

### Eurema floricola leonis

(Butler, 1886)

D5D4E009-2809-5A93-A5AE-CB32992A5ABF

#### Materials

- **Type status:** Other material. **Occurrence:** individualCount: 1; lifeStage: adult; **Taxon:** scientificName: Eurema floricola leonis (Butler, 1886); taxonRank: subspecies; scientificNameAuthorship: (Butler, 1886); **Location:** continent: Africa; country: Cameroon; stateProvince: Southwest Region; locality: Radio Hill, Bimbia village; verbatimElevation: 220 m; decimalLatitude: 3.9666; decimalLongitude: 9.2411; **Identification:** identifiedBy: Szabolcs Sáfián | Robert Tropek; dateIdentified: 2015; **Event:** samplingProtocol: Butterfly net; eventDate: 05/09/2015; habitat: Hilltop with disturbed lowland forest; **Record Level:** type: PhysicalObject; institutionID: http://grbio.org/cool/8t1f-g2z6; institutionCode: ZMJU; basisOfRecord: PreservedSpecimen
- **Type status:** Other material. **Occurrence:** individualCount: 1; lifeStage: adult; **Taxon:** scientificName: Eurema floricola leonis (Butler, 1886); taxonRank: subspecies; scientificNameAuthorship: (Butler, 1886); **Location:** continent: Africa; country: Cameroon; stateProvince: Southwest Region; locality: Mundemba; verbatimElevation: 20 m; decimalLatitude: 4.93; decimalLongitude: 8.854; **Identification:** identifiedBy: Robert Tropek; dateIdentified: 2015; **Event:** samplingProtocol: Butterfly net; eventDate: 01/02/2010; habitat: Disturbed secondary regrowths; **Record Level:** type: PhysicalObject; institutionID: http://grbio.org/cool/06zb-203s; institutionCode: IECA; basisOfRecord: PreservedSpecimen
- **Type status:** Other material. **Occurrence:** individualCount: 1; lifeStage: adult; **Taxon:** scientificName: Eurema floricola leonis (Butler, 1886); taxonRank: subspecies; scientificNameAuthorship: (Butler, 1886); **Location:** continent: Africa; country: Cameroon; stateProvince: Centre Region; locality: Ebogo Forest; verbatimElevation: 660 m; decimalLatitude: 3.388; decimalLongitude: 11.47; **Identification:** identifiedBy: Robert Tropek; dateIdentified: 2015; **Event:** samplingProtocol: Butterfly net; eventDate: 01/12/2008; habitat: Disturbed lowland forest; **Record Level:** type: PhysicalObject; institutionID: http://grbio.org/cool/06zb-203s; institutionCode: IECA; basisOfRecord: PreservedSpecimen
- **Type status:** Other material. **Occurrence:** individualCount: 1; lifeStage: adult; **Taxon:** scientificName: Eurema floricola leonis (Butler, 1886); taxonRank: subspecies; scientificNameAuthorship: (Butler, 1886); **Location:** continent: Africa; country: Cameroon; stateProvince: South Region; locality: Ebodje; verbatimElevation: 20 m; decimalLatitude: 2.572; decimalLongitude: 9.832; **Identification:** identifiedBy: Robert Tropek; dateIdentified: 2015; **Event:** samplingProtocol: Butterfly net; eventDate: 12/17/2011; habitat: Secondary lowland forest; **Record Level:** type: PhysicalObject; institutionID: http://grbio.org/cool/06zb-203s; institutionCode: IECA; basisOfRecord: PreservedSpecimen

#### Distribution

**Figure 33.**
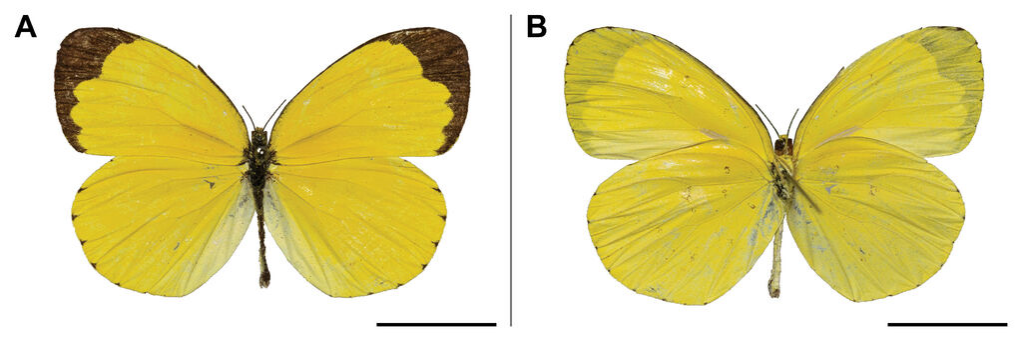
*Eurema floricola leonis* (Butler, 1886). **A.** dorsal view; **B.** ventral view. The scale bar represents 1 cm.

This species was recorded from West Africa (from Guinea-Bissau to Nigeria) to Central (Democratic Republic of the Congo) and Eastern Africa. Especially because it was already known from the Cross River State in Nigeria (Larsen 2005), its occurrence in Cameroon could be expected. We collected four specimens, each in a different disturbed lowland forest (from 20 to 660 m a.s.l.), therefore the species seems to be widespread, but locally scarce in Cameroon. This fully corresponds with T. Larsen’s experience with this species in Nigeria (Larsen 2005) (Fig. 33).

## Discussion

Altogether, the Lepidoptera species included in this study comprise 31 species and 8 genera new for the entomofauna of Cameroon, as well as a butterfly *T. encedana* that had not been recorded for more than a century in Cameroon. With the records from this study, the known diversity in Cameroon now surpasses 3,000 taxa for moths (species and subspecies combined; De Prins and De Prins 2019), whilst it approximates 1,600 taxa for butterflies (Williams 2018).

Of the 31 species new for Cameroon, four are known from the Guinean biogeographic region only (De Prins and De Prins 2019, Williams 2018). Therefore, our records did not change their endemic status, although this study extended their distribution to the easternmost border of the Guinean region (including an extension of ca. 1,000 km for *A. holobrunnea* and *A. macaria*). Another ten of the listed species had been previously recorded in the Congolian region only. From these, our records have extended distribution of *Euphaedra temeraria* northwards, while the other species’ distributions have been extended eastwards (De Prins and De Prins 2019, Williams 2018). Mainly, we have evidenced these ten species to occur in the Guinean biogeographic region, although only at its edge. This broadening of the easternmost, westernmost or northernmost limits of the numerous species distribution shows the importance of Cameroon (with special emphasis on Mount Cameroon) as the ‘crossroads’ between the Guinean and the Congolian biogeographic regions. It also reinforces Mount Cameroon as a biodiversity hotspot area (Myers et al. 2000, Ustjuzhanin et al. 2018).

Several other species records included in this report are not surprising since they more or less expectedly fill gaps in their known distribution. Most expectedly, we recorded twelve species (such as *A. makomensis* and *S. plagiatus*) already known from the countries bordering Cameroon or some other nearby countries in the Guinean and Congolian regions (De Prins and De Prins 2019). Six other species (such as *P. spurrelli* and *M. tripunctata*) were known to have a more scattered distribution amongst West, East and South Africa, but in countries more distant from Cameroon, forming a relatively large gap in their known distribution. Aside from the species with azonal distribution (such as the montane species), such large gaps rather imply lack of knowledge on the species occurrence (cf. Maicher et al. 2016). Our records in Cameroon have confirmed such suggestion by the partial filling of these distribution gaps. The record of the widespread *T. encedana* more than 100 years after its first and only record in Cameroon is also a perfect illustration of this general lack of knowledge on the Cameroonian (and Afrotropical) biodiversity of Lepidoptera. This was already pointed out by Tropek et al. (2013) and Tropek et al. (2015) for butterflies and by Maicher et al. (2016) for moths.

Considering the elevational ranges of the reported species, 19 reported lepidopteran species were exclusively collected in lowland and upland forests (between 30 and 1,100 m a.s.l) on Mount Cameroon, while records of five moth species were restricted to submontane and montane forests (between 1,450 and 2,100 m a.s.l.). Consequently, given that the local lepidopteran diversity is known to be higher at lower elevations (differing amongst the lepidopteran groups but always up to 1,100 m a.s.l.; Maicher et al. 2020), it appears that the knowledge gap seems proportionally comparable between the lower and higher elevations on the mountain. Although knowledge on precise elevational ranges of Afrotropical moths is highly limited, all five high-elevation species were previously reported from countries with montane ranges. These species are also the ones with the known azonal distributions which can be related to their affiliation to (sub)montane habitats rather than the severe lack of knowledge on their actual distribution. The remaining seven species have been recorded in both lowland and montane forests on Mount Cameroon.

In conclusion, our report of numerous butterfly and moth species and genera, previously not known to occur in Cameroon, highlights the relatively poor knowledge on the local and regional diversity of Afrotropical Lepidoptera. Moreover, some of these records represent a substantial extension of the individual species’ known distribution. Altogether, we encourage the collection and publishing of similar faunistic data on lepidopteran occurrence from the Afrotropical countries.

## Supplementary Material

XML Treatment for Anapisa holobrunnea

XML Treatment for Anapisa metarctioides

XML Treatment for Archithosia makomensis

XML Treatment for Balacra compsa

XML Treatment for Daphaenisca inexpectata

XML Treatment for Hippurarctia judith

XML Treatment for Ligulosia costimaculata

XML Treatment for Palaeugoa spurrelli

XML Treatment for Calligraphidia opulenta

XML Treatment for Uripao albizonata

XML Treatment for Dasychira punctifera

XML Treatment for Euproctis ceramozona

XML Treatment for Lomadonta saturata

XML Treatment for Orgyia basalis

XML Treatment for Pirga ubangiana

XML Treatment for Rhypopteryx rubripunctata

XML Treatment for Stenoglene plagiatus

XML Treatment for Hypotrabala castanea

XML Treatment for Mimopacha tripunctata

XML Treatment for Pachytrina gliharta

XML Treatment for Archinadata aurivilliusi

XML Treatment for Brachychira punctulata

XML Treatment for Gargettoscrancia albolineata

XML Treatment for Pseudobarobata denticulata

XML Treatment for Pseudobarobata integra

XML Treatment for Borbo borbonica

XML Treatment for Meza mabillei

XML Treatment for Acraea macaria

XML Treatment for Telchinia encedana

XML Treatment for Euphaedra temeraria

XML Treatment for Neptis metella

XML Treatment for Eurema floricola leonis

## Figures and Tables

**Figure 2a.**
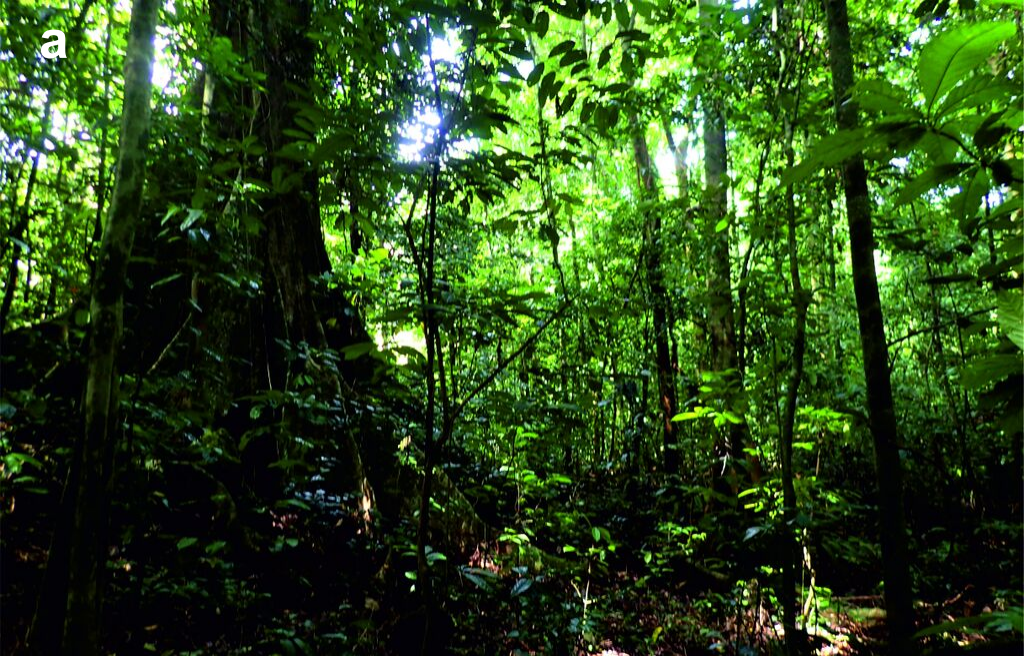
Coastal forest in the Bimbia-Bonadikombo Community Forest, Dikolo Peninsula (30 m a.s.l.).

**Figure 2b.**
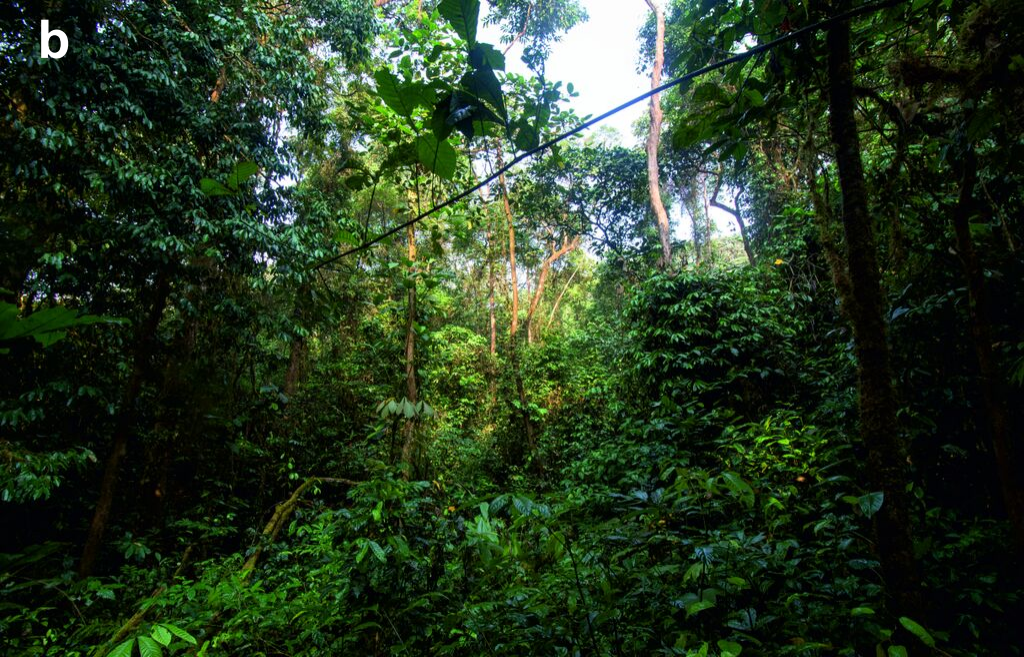
Primary lowland forest, Drink Gari, Mount Cameroon (650 m a.s.l.).

**Figure 2c.**
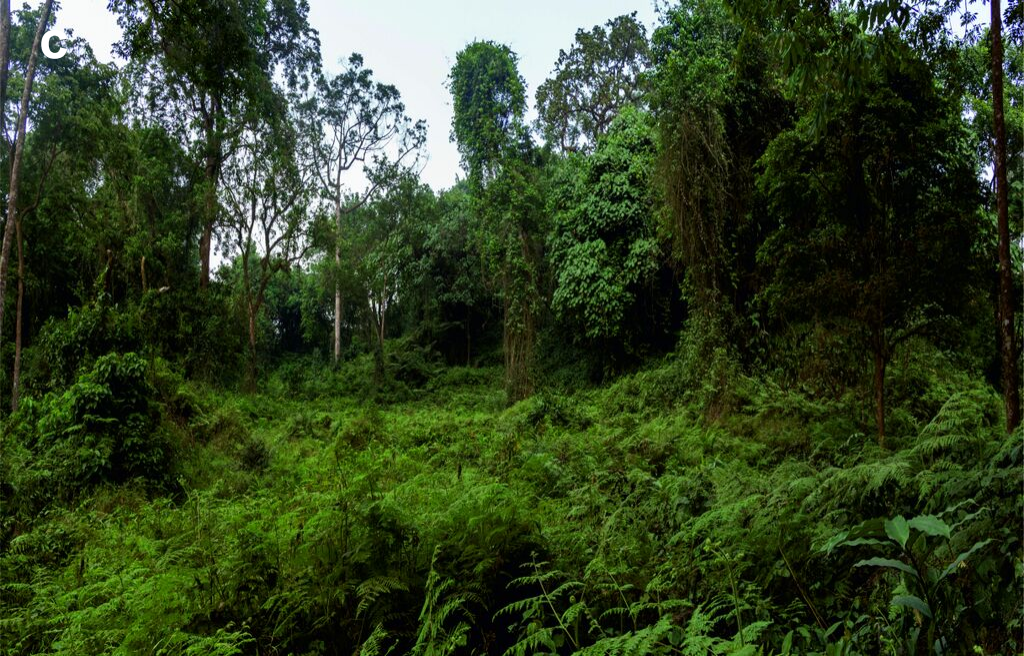
Upland forest locally disturbed by elephants, PlanteCam Camp, Mount Cameroon (1,100 m a.s.l.).

**Figure 2d.**
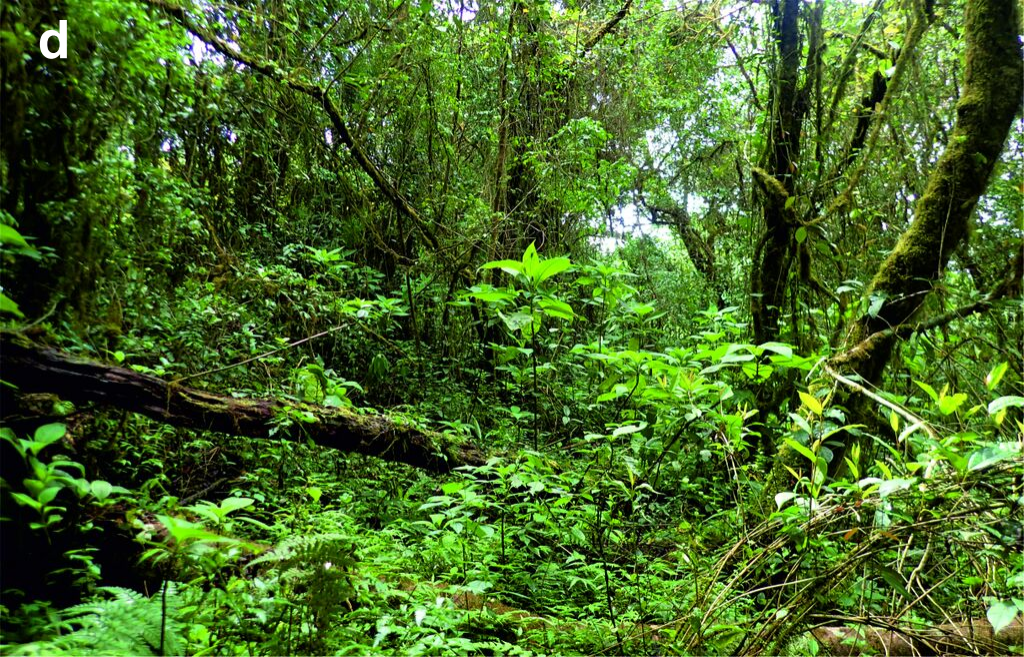
Montane forest, Mapanja camp, Mount Cameroon (1,850 m a.s.l.)

**Figure 2e.**
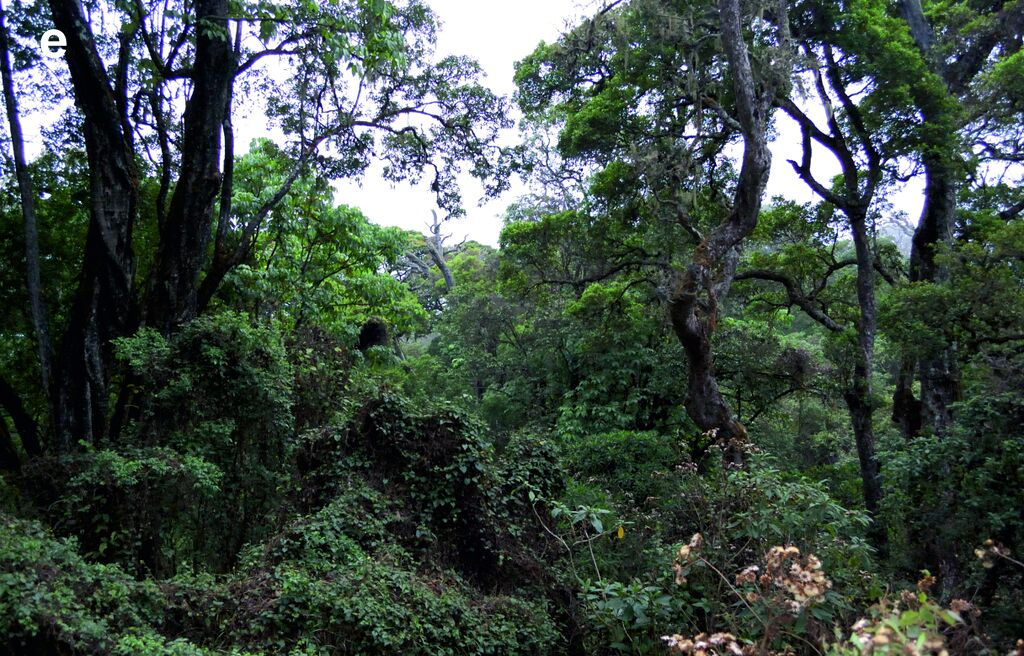
Montane forest close to the timberline, Mann’s Spring, Mount Cameroon (2,200 m a.s.l.).

**Figure 2f.**
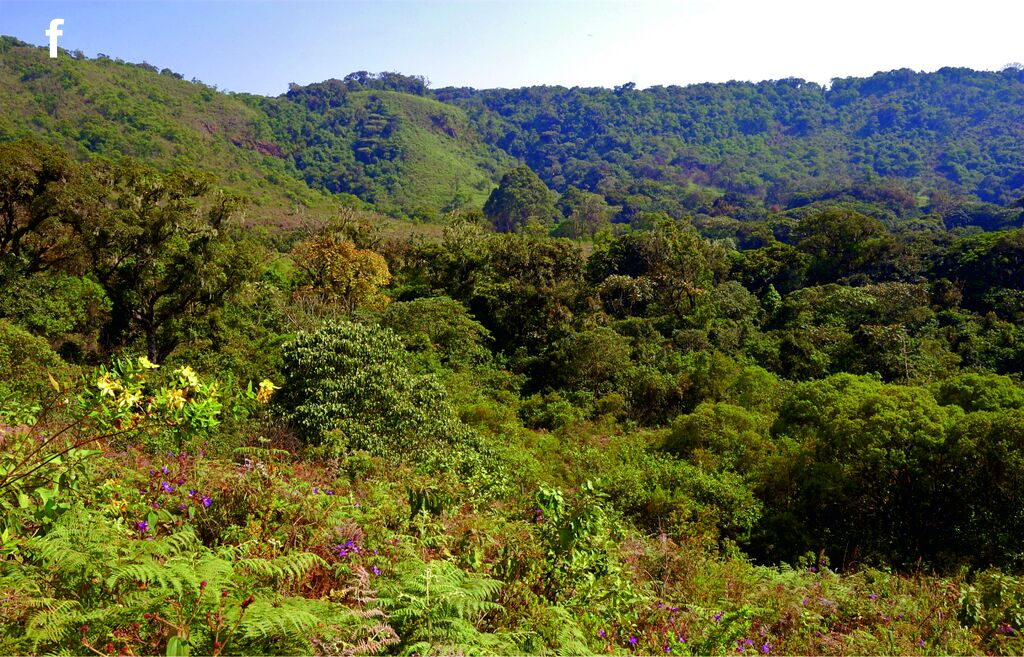
Mosaic of montane forest remnants and open habitats in Mendong Buo (2,200 m a.s.l.).
